# Use of Continuous Glucose Monitors in Exercise Research Studies—A Scoping Review on Study Characteristics and Common Practices

**DOI:** 10.3390/sports14070274

**Published:** 2026-07-02

**Authors:** Leon Schwensfeier, Christian Brinkmann

**Affiliations:** 1 Department of Preventive and Rehabilitative Sport Medicine, Institute of Cardiovascular Research and Sport Medicine, German Sport University Cologne, Am Sportpark Müngersdorf 6, 50933 Cologne, Germany; l.schwensfeier@dshs-koeln.de; 2 Department of Fitness & Health, IST University of Applied Sciences, Erkrather Straße 220, 40233 Düsseldorf, Germany

**Keywords:** CGM, continuous glucose monitoring, physical activity, exercise, training

## Abstract

This review examined study characteristics and common practices in continuous glucose monitoring (CGM)-based exercise studies. Following PRISMA guidelines, a systematic search in the PubMed database was conducted. Data were extracted from 93 publications. A minority of studies (12.9%) focused on CGM system validation. Acute exercise studies were more common (87.1%) than chronic exercise studies (12.9%). Randomized crossover designs predominated (71.0%). Participant populations varied and included 46.2% non-diabetic individuals (7.5% athletes), 36.6% individuals with type 1 diabetes mellitus, and 17.2% individuals with type 2. The upper arm was the most common sensor placement site (41.9%), although nearly one-third of studies did not report placement details. Devices were primarily from Abbott (40.9%), Medtronic (36.6%), and Dexcom (26.9%). Sample sizes were typically small, with 40.9% of studies including 10–14 participants. Reporting practices frequently deviated from the International Consensus Statement on CGM Metrics for Clinical Trials. Many studies used modified or non-standard metrics, whereas “mean sensor glucose” was reported in compliance with consensus recommendations in 58.1% of studies. Regarding data completeness, “data gaps” was the most frequently reported consensus-compliant metric (43.0%). In validation studies, accuracy metrics predominated, with “absolute relative difference” representing the most common outcome (87.5%). Overall, substantial heterogeneity limits comparability across studies, highlighting the need for standardized CGM reporting.

## 1. Introduction

Continuous glucose monitoring (CGM) systems were originally developed for the management of diabetes mellitus [1,2,3]. The use of CGM devices has increased significantly in recent years [4,5], and their benefits have been well documented for patients with both type 1 diabetes mellitus (T1DM) and type 2 diabetes mellitus (T2DM) [6,7]. Interest in CGM has also increased among non-diabetic populations for a range of possible lifestyle applications, including improving dietary habits, increasing physical activity and optimizing athletic performance [2,8,9,10]. This growing interest in CGM technology is also reflected in academic research by a notable increase in CGM-based publications [11,12].

Given the wide range of potential applications across different population groups, it is reasonable to assume some heterogeneity in how research studies use CGM systems and report results. In this context, standardized CGM outcomes and reporting frameworks have already been established, such as the “Ambulatory Glucose Profile” (AGP) [13] or the International Consensus Statement on CGM and Metrics for Clinical Trials [14]. In addition, there are recommendations for reporting clinical performance evaluations of CGM systems [15].

This scoping review aims to examine study characteristics and common practices in CGM-based exercise research and to assess whether the reporting of CGM metrics aligns with current expert recommendations.

## 2. Materials and Methods

### 2.1. Review Protocol and Literature Search

A scoping review protocol was developed in accordance with PRISMA guidelines for scoping reviews [16]. Given the iterative nature of scoping reviews, which may require methodological refinements as familiarity with the evidence base evolves, we did not prospectively register a protocol for this review. Eligible studies involved the use of CGM in the context of (partially or fully) supervised acute or chronic exercise interventions with detailed information on exercise modalities (volume, frequency, intensity) and included adults aged ≥ 18 years. Review articles, meta-analyses, study protocols, observational studies, or secondary analyses were excluded. Studies based exclusively on unsupervised home exercise, educational, counseling, or motivational interventions aimed at promoting exercise, the provision of exercise videos, or physical activity without a structured intervention were also excluded. The literature search was conducted in the PubMed database, as it provides comprehensive coverage of biomedical and exercise science literature. The literature search was limited to studies published between 1 January 2019 and 1 February 2025. Studies published before 1 January 2019 were excluded to ensure that the review reflects current CGM technology, as rapid technological advancements may limit the comparability of older and more recent studies. The following search string was used: *(CGM OR “continuous glucose monitor”) AND (exercise OR training OR sport)*.

The retrieved records were managed in *Rayyan* (Rayyan Systems Inc., Cambridge, MA, USA), a web-based platform for systematic literature reviews. Duplicates were removed prior to screening. The screening process was conducted in two stages: First, titles and abstracts were reviewed, followed by a review of the full texts. A human reviewer (LS) assessed all records against the predefined eligibility criteria. In addition, the OpenAI o1 model, accessed through ChatGPT (OpenAI OpCo, LLC, San Francisco, CA, USA) [17], was used as an assistive tool to classify each record as “include”, “exclude”, or “maybe”. For this purpose, a predefined prompt (Supplementary Materials File S1: Prompt) specifically tailored to the screening process and aligned with the eligibility criteria was applied. To improve output quality, the prompt was optimized for academic purposes through prompt engineering techniques [18]. A second reviewer (CB) reviewed only those cases in which the decisions of LS and ChatGPT differed, as well as records classified as “maybe”. LS and CB discussed these cases until consensus was reached. Final decisions regarding study inclusion or exclusion were made by the human reviewers, with ChatGPT’s classifications serving solely to support the screening process.

### 2.2. Data Extraction

Prior to the full data extraction, the authors developed and tested a standardized data extraction template to ensure consistent data collection. One of the authors (LS) manually extracted the following study characteristics and CGM outcomes from the included studies: study objective (validation, non-validation), intervention duration (acute or chronic exercise), study design, study population, sample size, sensor placement site, CGM system(s) used (manufacturer/company), reported CGM metrics, and metrics on data completeness as well as reported CGM performance metrics. The precise definitions of the categories developed for the extracted study characteristics and CGM outcomes are provided below:

*Primary objectives*: The studies were classified according to their primary objective as stated by the authors (in the title, abstract, introduction, or methods section): non-validation studies were defined as those primarily aimed at investigating glucose dynamics under various conditions, rather than at validating a CGM system. Validation studies were defined as those primarily aimed at evaluating the accuracy or technical reliability of CGM systems, rather than at examining glucose dynamics under various conditions.

*Acute or chronic exercise studies*: Acute exercise studies were defined as those in which the longest consecutive exercise block spanned 7 calendar days and consisted of <8 exercise sessions. Chronic exercise studies were defined as those in which the longest consecutive exercise block spanned > 7 calendar days or consisted of ≥8 exercise sessions. Studies involving monitoring, washout, and run-in phases without exercise were not considered part of the intervention period. In multi-arm and crossover studies, each arm was classified separately. Each study as a whole was classified as an acute exercise study only if all arms met the criteria for acute exercise exposure, and as chronic exercise studies if at least one arm met the criteria for chronic exercise exposure.

*Study designs:* The study designs were classified according to the following coding criteria: a randomized crossover was defined as a within-subjects design in which the same participants completed two or more different intervention phases in a randomized order, with outcomes compared across phases, such that each participant served as their own control. A single-group non-crossover design was defined as a study in which all participants received the same intervention and could be measured repeatedly across time points, conditions, sensor sites, devices or phases, without parallel group allocation and without sequential alternation between different intervention phases for the same individual. A randomized parallel-group design was defined as a between-group design in which participants were randomly assigned to two or more study arms and remained in a single arm for the duration of the intervention comparison. A non-randomized crossover design was defined as a within-subjects design in which the same participants underwent two or more intervention phases in a fixed, non-randomized order, with outcomes compared across phases. A non-randomized parallel-group design was defined as a between-group design in which participants were assigned to two or more study arms without randomization and remained in a single arm for the intervention comparison.

*Study populations:* Study participants were grouped according to the target population defined in each studies’ inclusion criteria and baseline characteristics: non-diabetic referred to healthy participants without a diabetes diagnosis, while T1DM/T2DM referred to participants diagnosed with the respective condition. Healthy athletes referred to participants who were professional, competitive, or recreational athletes engaging in specific sports (e.g., runners, cyclists), while excluding normally active participants. Other participants included samples that could not be assigned to a single category, such as mixed groups (e.g., participants with T2DM or prediabetes), athletes with T1DM, and pancreatic islet transplant recipients.

*Sensor placement sites:* The CGM sensor application site was coded based on information provided by the authors. Multiple entries were permitted, as some studies examined multiple sensor sites. If no explicit information on sensor placement was provided, the application site was inferred only when it could be unambiguously identified based on the clearly specified CGM system and the manufacturer-approved application site(s). Otherwise, the placement site was recorded as “Not stated”.

*CGM systems used:* The CGM manufacturer was coded based on the authors’ explicit reporting. If the manufacturer was not explicitly stated, it was inferred only when the specified sensor or CGM system model could be unambiguously assigned to a single manufacturer. Otherwise, the manufacturer was coded as “Not stated”.

*Sample sizes:* The actual sample size analyzed was recorded. Where different sample sizes were reported across study arms, endpoints or time points, the largest analyzed sample size was used. To plot the sample size, the class width of the diagram was calculated using the Freedman–Diaconis rule [19]. The resulting width (≈4.62 participants) was rounded to five participants for easier interpretation. The theoretical number of classes (k) was then estimated using $k\approx \frac{\max-min}{h}$, which yielded k = 13. However, since the upper range of the distribution was sparsely represented, the classes above 35–39 were merged into a single category (i.e., ≥40).

*Reported CGM metrics:* All CGM-related metrics explicitly reported in the results sections of the included studies were extracted. Consequently, metrics that were described only in the methods section, but were not reported in the results section were not considered. The reported CGM metrics were classified according to categories defined in existing consensus statements and recommendations. Accordingly, CGM metrics such as time in range (TIR), time above range (TAR) and time below range (TBR) were categorized in line with the International Consensus Statement on CGM and Metrics for Clinical Trials [14], while CGM performance parameters and methods such as mean absolute relative difference (MARD), the Bland–Altman plot, and the Clarke error grid (CEG), were categorized according to the Recommendations for Reporting Clinical Performance Evaluations of CGM Systems [15]. To improve the data’s interpretability, outcomes were extracted without time specifications. For example, although Battelino et al. [14] recommend reporting glucose data derived from CGM sensors separately for nighttime (00:00 to 05:59) and daytime (06:00 to 23:59), these temporal distinctions were not considered during data extraction. The extracted outcomes were further evaluated for compliance with the respective consensus statement or recommendations. For CGM metrics [14], the following coding criteria were applied: mean sensor glucose (SG) was coded as compliant only when reported as a single mean value, and as non-compliant when reported exclusively as a time series, a median value, or not reported at all. The coefficient of variation (CV) and standard deviation (SD) of mean SG were coded as compliant if reported as a single numerical value and/or graphically, and as non-compliant if omitted. The TBR, TAR and TIR metrics were coded as compliant only when reported using the exact consensus-defined thresholds, and as non-compliant if alternative cutoff values were used, no thresholds were specified, or the endpoint was omitted. 

*Reported data completeness metrics:* For data completeness metrics, the percentage of sensor data obtained was coded as compliant when reported as the percentage of recorded CGM readings relative to the expected number of readings. Data gaps were coded as compliant if any quantitative and/or qualitative information was provided; frequency of scanning was recorded as compliant if reported quantitatively (e.g., scans/day, scan interval as h/min per day/intervention phase) and/or qualitatively (e.g., instructions to scan every 8 h), but this criterion was only applied to studies using intermittently scanned CGM (isCGM) systems.

*Reported CGM performance metrics:* As regards CGM performance parameters [15], technical reliability was classified according to the following criteria: device deficiencies were coded as compliant when information on possible malfunctions affecting proper system functioning were reported (e.g., hardware/software errors, scanning failures, signal loss, or adverse events such as skin reactions), while excluding accidental physical detachment of sensors. Sensor survival was coded as compliant when information was provided on whether sensors reached their intended wear duration, including numerical survival data or percentages, as well as reports of premature sensor loss (e.g., detached sensors). Data availability was coded as compliant when the number of recorded glucose readings was reported as a percentage or ratio of the expected number of glucose readings, and time-lag analysis was coded as compliant if any quantitative method of estimation was reported, whereas general discussions of lag time alone were not considered sufficient.

In addition to the manual data extraction performed by LS, the GPT-5.4 Thinking model, accessed through ChatGPT (OpenAI OpCo, LLC, San Francisco, USA) [20], was used as a supportive tool to extract data from full-text articles using a predefined prompt (Supplementary Materials File S2: Prompt). All data extracted by ChatGPT were compared with the manually extracted data to identify potential extraction errors. Any discrepancies were resolved by re-examining the full-text article by LS.

The extracted data were summarized descriptively and presented using bar and pie charts. All figures were created using Microsoft Excel (Microsoft 365, Microsoft Corporation, Redmond, WA, USA).

## 3. Results

**Figure 1:**
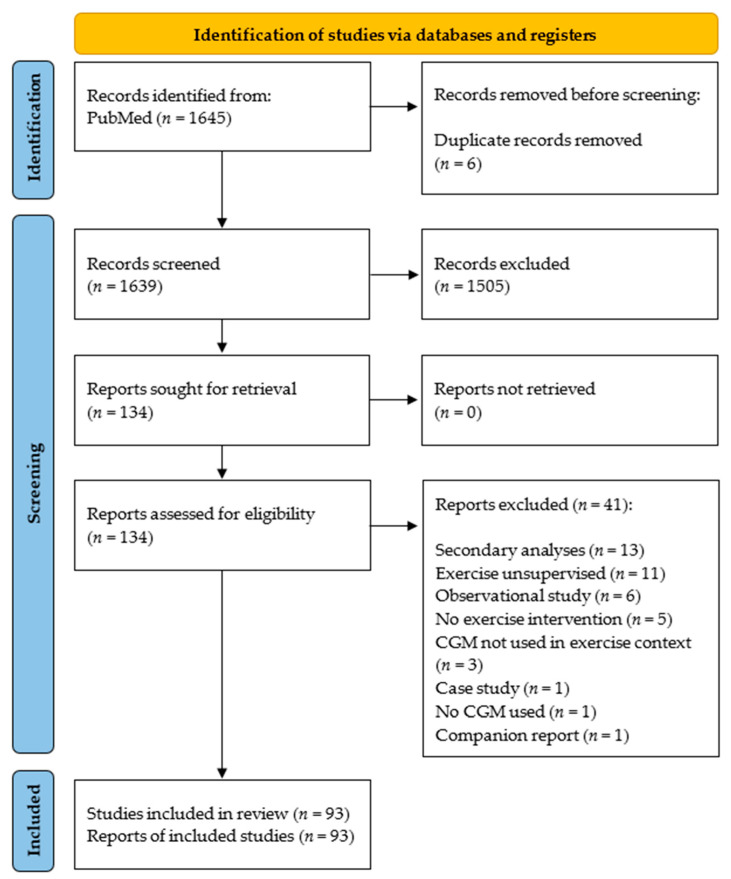
PRISMA Flow Diagram.

The literature search and study selection process are illustrated in Figure 1. The search identified *n* = 1645 articles, of which *n* = 1639 remained after the removal of duplicates. The human reviewer (LS) classified 1439 records as “to be excluded” following title and abstract screening, whereas ChatGPT [17] classified 1519 records as “to be excluded”. After the human reviewers (LS and CB) reached consensus on these discrepancies and all “maybe” cases, a total of *n* = 1505 records were excluded, and *n* = 134 articles were retained for full-text analysis. After full-text review, *n* = 41 articles were excluded. Finally, *n* = 93 studies [21,22,23,24,25,26,27,28,29,30,31,32,33,34,35,36,37,38,39,40,41,42,43,44,45,46,47,48,49,50,51,52,53,54,55,56,57,58,59,60,61,62,63,64,65,66,67,68,69,70,71,72,73,74,75,76,77,78,79,80,81,82,83,84,85,86,87,88,89,90,91,92,93,94,95,96,97,98,99,100,101,102,103,104,105,106,107,108,109,110,111,112,113] were included in the analysis (see the list of publications in Supplementary Materials File S3: Table and all extracted data in Supplementary Materials File S4: Coded Outcomes).

### 3.1. Study Objectives

**Figure 2:**
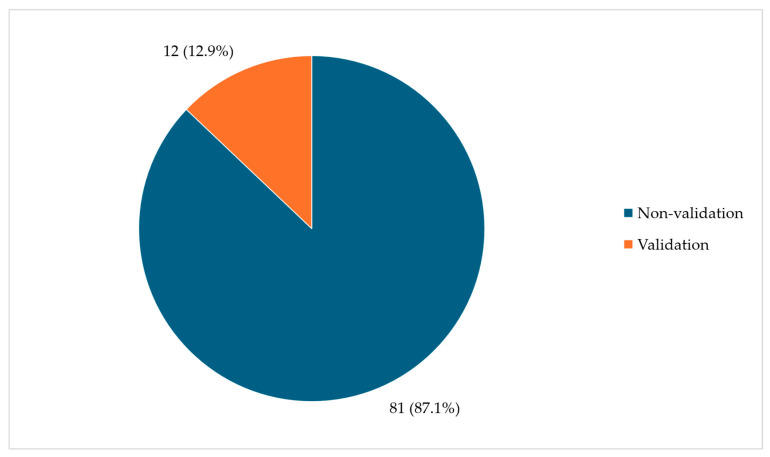
Reported Main Study Objective in Included Studies. The figure shows the distribution of study objectives across the 93 included studies. As each study contributes exactly one entry, segments represent the number of studies within each category and their corresponding percentage relative to all included studies.

Most included studies focused on analyzing glucose responses under various exercise conditions (non-validation studies), while a smaller proportion of studies aimed to validate CGM systems (Figure 2). Although validation was not the primary objective, four studies reported CGM performance parameters in addition to clinical outcomes.

### 3.2. Acute or Chronic Exercise Studies

**Figure 3:**
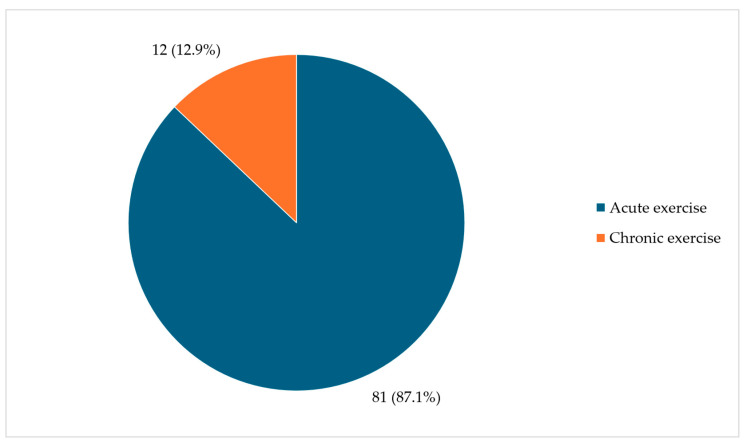
Reported Study Information on Acute or Chronic Exercise in Included Studies. The figure shows the distribution of acute and chronic exercise studies across the 93 included studies. As each study contributes exactly one entry, segments indicate the number of studies within each category and their corresponding percentage relative to all included studies.

The proportion of included studies focusing on acute exercise using CGM was higher than the proportion of studies examining the long-term effects of physical activity (Figure 3).

### 3.3. Study Designs

**Figure 4:**
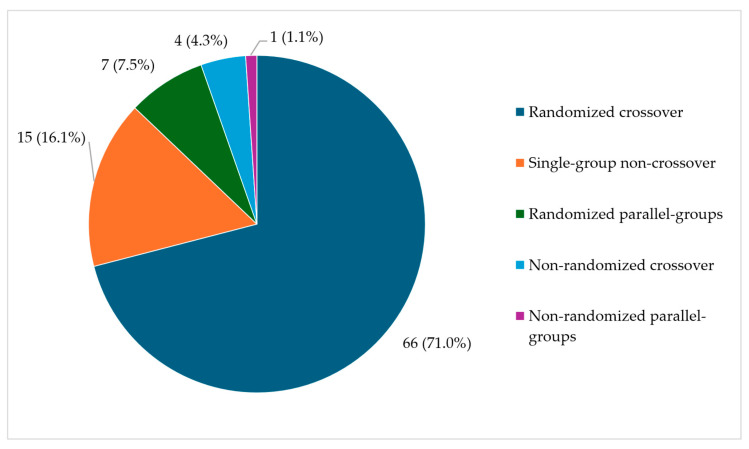
Reported Study Design in Included Studies. The figure shows the distribution of study designs across the 93 included studies. As each study contributes exactly one entry, segments indicate the number of studies within each category and their corresponding percentage relative to all included studies.

As illustrated in Figure 4, the most frequently used study designs among the included studies involved within-subject comparisons, with randomized crossover studies accounting for the largest proportion.

### 3.4. Study Populations

**Figure 5:**
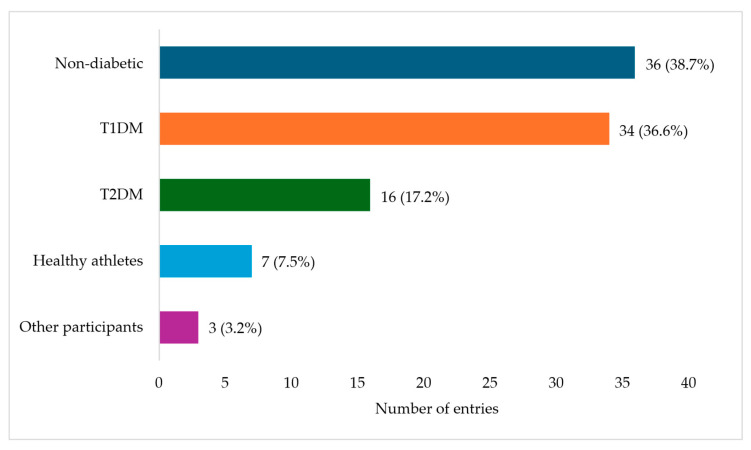
Reported Participant Population in Included Studies. The figure shows the distribution of participant populations across the 93 included studies. Bars represent the number of entries per category and their corresponding percentage relative to all included studies. As studies could contribute multiple population entries, the total number of entries (i.e., 96) exceeds the number of included studies.

Figure 5 shows that the included studies primarily examined non-diabetic individuals and people with T1DM. “Healthy athletes” were considered separately from the “Non-diabetic” group. “Healthy athletes” referred to participants who were professional, competitive, or recreational athletes. Study populations that could not be assigned to a single category, including mixed participant groups, were classified as “Other participants”.

### 3.5. Sample Sizes

**Figure 6:**
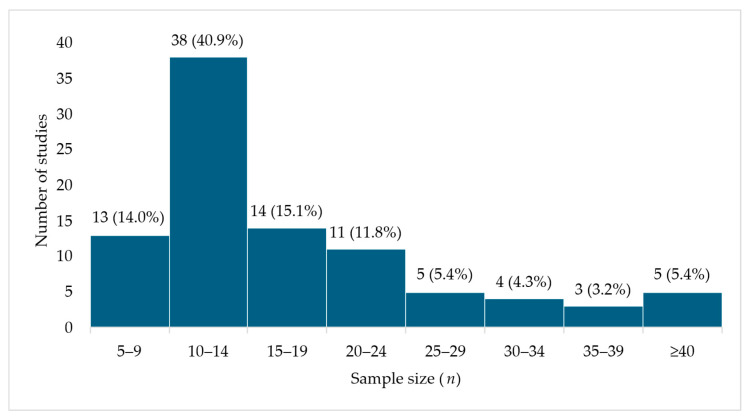
Reported Study Sample Size in Included Studies. The figure shows the sample sizes across the 93 included studies. As each study contributes exactly one entry, bars represent the number of studies within each category and their corresponding percentage relative to all included studies. The group “≥40 participants” comprises sample sizes of 48, 50, 56, 60 and 63.

As illustrated in Figure 6, most of the included studies had a relatively small sample size (mostly 10–14 participants). Studies with larger sample sizes were relatively rare.

### 3.6. Sensor Placement Sites

**Figure 7:**
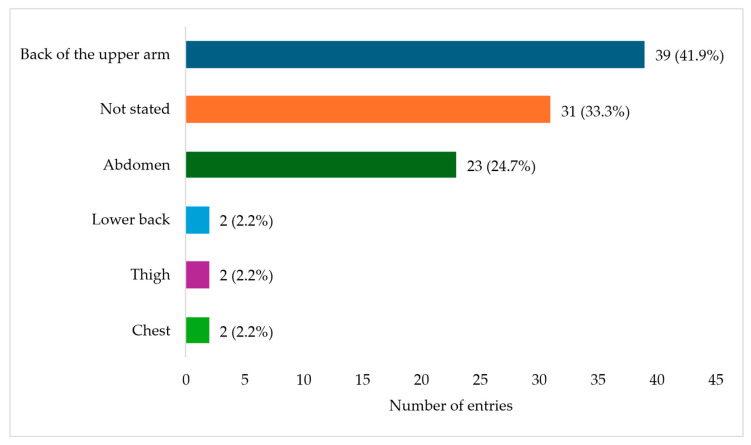
Reported CGM Sensor Application Site in Included Studies. The figure shows the distribution of sensor application sites across the 93 included studies. Bars represent the number of entries within each category and their corresponding percentage relative to all included studies. As studies could contribute multiple application site entries, the total number of entries (i.e., 99) exceeds the number of included studies.

Among the included studies, CGM sensors were most commonly attached to the back of the upper arm or abdomen (Figure 7). In nearly one-third of the studies, the authors did not specify the attachment site.

### 3.7. CGM Systems Used

**Figure 8:**
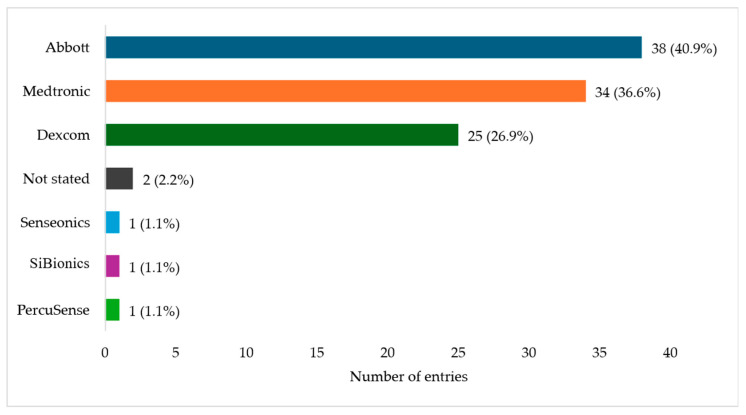
Reported CGM System (Company) in Included Studies. The figure shows the distribution of the CGM systems’ manufacturers across the 93 included studies. Bars represent the number of entries within each category and their corresponding percentage relative to all included studies. As studies could contribute multiple CGM system company entries, the total number of entries (i.e., 102) exceeds the number of included studies.

Six CGM manufacturers were identified among the CGM systems used in the included studies. Their official company names, abbreviated in Figure 8, were Abbott Diabetes Care Inc. (Alameda, CA, USA), Medtronic plc (Dublin, Ireland), Dexcom Inc. (San Diego, CA, USA), Senseonics Holdings, Inc. (Germantown, MD, USA), Shenzhen SiSensing Co., Ltd. (SiBionics; Shenzhen, Guangdong, China), and PercuSense, Inc. (Valencia, CA, USA). Most studies used CGM systems manufactured by Abbott, Medtronic and Dexcom. In some cases, the authors did not provide information about the manufacturer of the CGM system used.

### 3.8. Reported CGM Metrics

**Figure 9:**
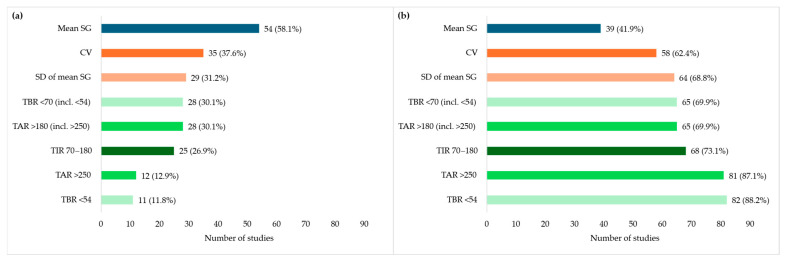
CGM-Derived Core Metrics for Clinical Trials Reported in Included Studies: Consensus-Based Classification. The figure shows the reporting of consensus-compliant (**a**) and non-consensus-compliant (**b**) CGM core metrics across the 93 included studies. As each study contributes exactly one entry per metric, bars represent the number of studies per category and their corresponding percentage relative to all included studies. “Non-compliant” studies include those in which relevant information was omitted, unclear, or not reported in a format consistent with the consensus-based coding criteria. SG = sensor glucose, CV = coefficient of variation (glucose variability), SD = standard deviation, TBR = time below range, TAR = time above range.

Of the 93 studies included, 21 studies (22.58%) did not report a core metric, and eight studies (8.6%) did not report any of the CGM outcomes recommended by the International Consensus Statement on CGM and Metrics for Clinical Trials [14]. Figure 9 illustrates the distribution of core metrics across the included studies and categorizes them according to their consistency with the consensus recommendations. Several studies reported outcomes using thresholds that deviated from the consensus definitions, including 14 studies (15.05%) for “time below range” (TBR), 10 studies (10.75%) for “time above range” (TAR), and 13 studies (13.98%) for “time in range” (TIR). Figure 9b also accounts for studies in which the outcome was not reported at all.

### 3.9. Reported Data Completeness Metrics

**Figure 10:**
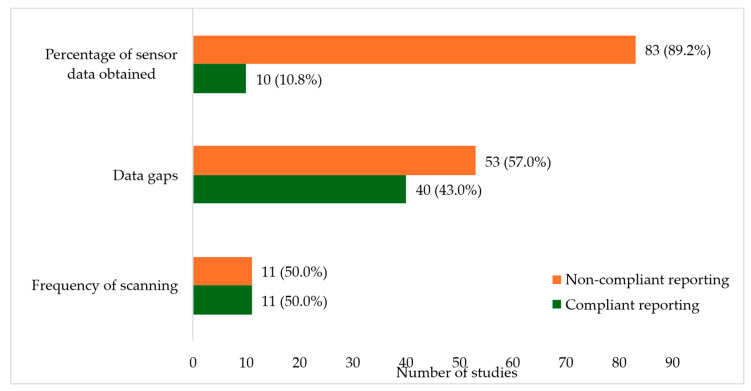
Data Completeness Metrics Reported in Included Studies: Consensus-Based Classification. The figure shows data completeness metrics reported across the 93 included studies. As each study contributes exactly one entry per metric, bars represent the number of studies per category and their corresponding percentage relative to all included studies. “Non-compliant” studies include those in which relevant information was omitted, unclear or not reported in a format consistent with the consensus-based coding criteria. The category “Frequency of scanning” was assessed only in studies using intermittently scanned CGM (isCGM; 22 studies), and percentages for this category are relative to these 22 studies.

Regarding data completeness, “Data gaps” was the most frequently reported consensus-compliant metric across studies (Figure 10). For at least half of the studies, reporting of individual data completeness metrics did not comply with the consensus recommendations.

### 3.10. Reported CGM Performance Metrics

**Figure 11:**
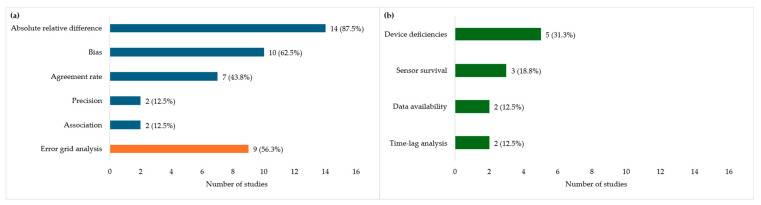
Recommendation-Compliant CGM Performance Metrics Reported in Included Studies. The figure shows the reporting of recommendation-compliant CGM performance metrics within the relevant subset of 16 studies that reported performance metrics. As each study contributes exactly one entry per metric, bars represent the number of studies per category and their corresponding percentage relative to all 16 studies in this subset. Panels depict point accuracy (**a**), comprising analytical (blue bars) and clinical point accuracy (orange bar), and technical reliability (**b**). The “Error grid analysis” category includes CG-EGA, which incorporates both point and trend accuracy.

Although only 12 studies were classified as validation studies, four additional non-validation studies also reported validation parameters. Accordingly, a total of 56 CGM performance outcomes, consistent with the overarching categories in the Recommendations for Reporting on Clinical Performance Evaluation of CGM [15] were reported across 16 studies (Figure 11). Reporting of point accuracy outcomes (Figure 11a) was incomplete across all six categories. More specifically, no data on “absolute relative difference (ARD)” were reported in two studies (12.5%), “bias” in six studies (37.5%), “agreement rate” in nine studies (56.2%), “precision” and “association” in 14 studies (87.5%), and “error grid analysis” in seven studies (43.7%).

Reporting of technical reliability metrics (Figure 11b) was also frequently absent, with no information on “device deficiencies” provided in 11 studies (68.7%), insufficient or no information reported on “data availability” in 14 studies (87.5%), no information provided on “sensor survival” in 13 studies (81.2%), and no information given on “time-lag analysis” in 14 studies (87.5%).

## 4. Discussion

Findings on study characteristics and common practices in CGM-based exercise studies may be relevant to researchers designing such studies, clinicians interpreting CGM-derived outcomes in different populations, or guideline developers.

The present scoping review, following the PRISMA guidelines (see Supplementary Materials File S5: PRISMA-ScR Checklist), shows that most studies using CGM devices in exercise interventions employed a randomized crossover design, included relatively small sample sizes of non-diabetic or T1DM participants, more frequently examined acute exercise rather than chronic exercise interventions, and primarily aimed to assess changes in glucose dynamics through exercise rather than validating CGM systems. The most commonly used CGM systems were manufactured by Abbott, Medtronic and Dexcom. The back of the upper arm and the abdominal area were the predominant sensor application sites.

Overall, reporting of CGM metrics was heterogeneous across all included studies [21,22,23,24,25,26,27,28,29,30,31,32,33,34,35,36,37,38,39,40,41,42,43,44,45,46,47,48,49,50,51,52,53,54,55,56,57,58,59,60,61,62,63,64,65,66,67,68,69,70,71,72,73,74,75,76,77,78,79,80,81,82,83,84,85,86,87,88,89,90,91,92,93,94,95,96,97,98,99,100,101,102,103,104,105,106,107,108,109,110,111,112,113], and in most cases, not aligned with current consensus statements and recommendations [14,15]. However, the current consensus recommendations were developed for diabetes care and clinical endpoints and may not necessarily be suitable for sports and exercise research or for the specific challenges in this field. These challenges include, for example, increased lag time between glucose values measured in blood and interstitial fluid, caused by rapid changes in blood glucose levels induced by physical activity [114,115,116,117], or sensor detachment and data loss due to sweating, movement, or swimming [26,118,119,120]. To better interpret the studies’ results and ensure comparability across studies, detailed information about the study procedures is required. Among other information, the sensor placement site should always be explicitly specified, as different application sites may yield different results. Coates et al. [34] investigated whether placing the sensor on the active muscle during cycling in non-diabetic individuals would yield different results compared with conventional placement on the arm. At rest, the leg sensor showed lower glucose values than the arm sensor and also following cycling with maximal intensity, ISF glucose measured in the leg was lower than in the arm. Mason et al. [70] studied non-diabetic, well-trained recreational runners and found that sensor accuracy was similar for arm and chest.

Furthermore, Battelino et al. [14] note that both the CGM brand and model used should be specified. The latter is particularly important because comparative measurements between devices from different manufacturers can show substantial differences in recorded values during physical activity [39,115].

Standardized reporting of CGM metrics is essential to ensure comparability of results across studies. However, the studies included in this review showed significant inconsistencies in CGM metric reporting. For example, studies that report TIR, TAR and TBR without specifying the corresponding thresholds and defined ranges are imprecise.

Regarding data completeness, Battelino et al. [14] recommend that at least 70% of potential CGM data over 14 consecutive days should be available. Our analysis indicates considerable gaps in the reporting of CGM metrics-related data quality/completeness across the included studies. In some cases, data completeness was inferred indirectly from the total time analyzed and the time lost due to signal failures [74]. Studies using intermittently scanned CGM (isCGM) systems—for which a minimum scanning frequency (i.e., three scans per day) and time interval (i.e., at least once every eight hours) are recommended for daily scans [14]—frequently exhibited reporting gaps. In fact, only half of the studies we analyzed and that used such systems reported scanning frequency in line with the recommendations. While it is possible that data completeness metrics were omitted in some studies because their datasets were indeed complete, it should be noted that explicit reporting is nevertheless essential to ensure data transparency and reliability.

Among the metrics used in the evaluation of CGM performance, analytical point accuracy was most frequently assessed, typically using MARD and/or bias analyses, often in combination with Bland–Altman plots. Overall, the findings suggest that reporting in validation studies is more consistent and in line with consensus recommendations than in non-validation studies.

Nevertheless, discrepancies between studies were also observed, such as in the reporting of agreement rates (defined as the proportion of CGM—blood glucose pairs falling within predefined limits). Although the ISO standard ISO 15197 [121] was developed for blood glucose meters, it is also sometimes used for reporting the accuracy of CGM systems. Matzka et al. [72] reported their data completely in line with the ISO 15197 requirements (at least 95% of results must be within ±15 mg/dL at glucose values < 100 mg/dL and within ±15% at glucose values ≥ 100 mg/dL). Elghobashy et al. [44] also reported both limits in accordance with ISO 15197, but reported the number of data points outside these limits rather than the resulting compliance percentages. Da Prato et al. [41] reported only data for glucose values ≥ 100 mg/dL within ±15%, as deviations in the remaining data were considered negligible, while Moser et al. [79] reported aggregated percentages of ISO 15197 compliance.

A limitation of this scoping review is that the literature search was conducted exclusively in PubMed. As a result, relevant studies from other databases may not have been identified. In addition, the consensus statements and recommendations used as reference standards in this review were primarily developed for diabetes care, clinical trials, or the evaluation of clinical performance. Therefore, they may not always be fully appropriate, particularly for CGM-based research projects in non-clinical settings.

## 5. Conclusions

Our analyses indicate that although several consensus statements and recommendations on CGM metric reporting and performance parameters exist [14,15], these are often not fully adhered to, and CGM outcome metrics are therefore frequently not reported in a clear, precise, and comparable manner across CGM exercise research studies. This heterogeneity in CGM outcome reporting coincides with differences in study characteristics, CGM systems used, sensor placement sites, and the reporting of data completeness. This presents challenges for synthesizing data and interpreting relevant CGM findings across studies. Future CGM studies in sports and exercise research should therefore specify the exact CGM system(s) and sensor placement site(s) used, comprehensively report consensus-compliant core metrics and/or CGM performance parameters, while also considering an extensive data completeness report. It would be valuable to develop specific guidelines for this field.

## Data Availability

All data discussed in this review are derived from previously published studies and are available in the cited references.

## Supplementary Materials

The following supporting information can be downloaded at: https://www.mdpi.com/article/10.3390/sports14070274/s1. File S1: Prompt Used for Eligibility Screening; File S2: Prompt Used for Data Extraction; File S3: Table of Publications Included in the Analyses (93 studies) [21,22,23,24,25,26,27,28,29,30,31,32,33,34,35,36,37,38,39,40,41,42,43,44,45,46,47,48,49,50,51,52,53,54,55,56,57,58,59,60,61,62,63,64,65,66,67,68,69,70,71,72,73,74,75,76,77,78,79,80,81,82,83,84,85,86,87,88,89,90,91,92,93,94,95,96,97,98,99,100,101,102,103,104,105,106,107,108,109,110,111,112,113]; File S4: Coded Outcomes [21,22,23,24,25,26,27,28,29,30,31,32,33,34,35,36,37,38,39,40,41,42,43,44,45,46,47,48,49,50,51,52,53,54,55,56,57,58,59,60,61,62,63,64,65,66,67,68,69,70,71,72,73,74,75,76,77,78,79,80,81,82,83,84,85,86,87,88,89,90,91,92,93,94,95,96,97,98,99,100,101,102,103,104,105,106,107,108,109,110,111,112,113]; File S5: Preferred Reporting Items for Systematic Reviews and Meta-Analyses Extension for Scoping Reviews (PRISMA-ScR) Checklist [16].

## References

[1] Gross T.M., Bode B.W., Einhorn D., Kayne D.M., Reed J.H., White N.H., Mastrototaro J.J. (2000). Performance evaluation of the MiniMed continuous glucose monitoring system during patient home use. Diabetes Technol. Ther..

[2] Holzer R., Bloch W., Brinkmann C. (2022). Continuous Glucose Monitoring in Healthy Adults-Possible Applications in Health Care, Wellness, and Sports. Sensors.

[3] U.S. Food and Drug Administration (2006). Summary of Safety and Effectiveness Data (SSED): DexCom STS Continuous Glucose Monitoring System (PMA No. P050012). https://www.accessdata.fda.gov/cdrh_docs/pdf5/p050012b.pdf.

[4] Atac O., Heier K.R., Moga D., Fowlkes J., Sohn M.W., Kruse-Diehr A.J., Waters T.M., Lacy M.E. (2025). Demographic variation in continuous glucose monitoring utilisation among patients with type 1 diabetes from a US regional academic medical centre: A retrospective cohort study, 2018–2021. BMJ Open.

[5] Pathak S., Kearin K., Kahkoska A.R., Fuller K.A., Staats B., Albright J., Stürmer T., Buse J.B., Urick B.Y. (2023). Impact of Expanding Access to Continuous Glucose Monitoring Systems Among Insulin Users with Type 1 or Type 2 Diabetes. Diabetes Technol. Ther..

[6] Ferreira R.O.M., Trevisan T., Pasqualotto E., Chavez M.P., Marques B.F., Lamounier R.N., van de Sande-Lee S. (2024). Continuous Glucose Monitoring Systems in Noninsulin-Treated People with Type 2 Diabetes: A Systematic Review and Meta-Analysis of Randomized Controlled Trials. Diabetes Technol. Ther..

[7] Klupa T., Czupryniak L., Dzida G., Fichna P., Jarosz-Chobot P., Gumprecht J., Mysliwiec M., Szadkowska A., Bomba-Opon D., Czajkowski K. (2023). Expanding the Role of Continuous Glucose Monitoring in Modern Diabetes Care Beyond Type 1 Disease. Diabetes Ther. Res. Treat. Educ. Diabetes Relat. Disord..

[8] Klonoff D.C., Nguyen K.T., Xu N.Y., Gutierrez A., Espinoza J.C., Vidmar A.P. (2023). Use of Continuous Glucose Monitors by People Without Diabetes: An Idea Whose Time Has Come?. J. Diabetes Sci. Technol..

[9] Oganesova Z., Pemberton J., Brown A. (2024). Innovative solution or cause for concern? The use of continuous glucose monitors in people not living with diabetes: A narrative review. Diabet. Med. A J. Br. Diabet. Assoc..

[10] Shah V.N., DuBose S.N., Li Z., Beck R.W., Peters A.L., Weinstock R.S., Kruger D., Tansey M., Sparling D., Woerner S. (2019). Continuous Glucose Monitoring Profiles in Healthy Nondiabetic Participants: A Multicenter Prospective Study. J. Clin. Endocrinol. Metab..

[11] Bevan A., Ellis G., Eskandarian M., Garrisi D. (2024). The Application of Continuous Glucose Monitoring Endpoints in Clinical Research: Analysis of Trends and Review of Challenges. J. Diabetes Sci. Technol..

[12] Kong L., Deng B., Guo M., Chen M., Wang X., Zhang M., Tang H., Wang Q., Yang L., Xiong Z. (2023). A systematic bibliometric analysis on the clinical practice of CGM in diabetes mellitus from 2012 to 2022. Front. Endocrinol..

[13] Bergenstal R.M., Ahmann A.J., Bailey T., Beck R.W., Bissen J., Buckingham B., Deeb L., Dolin R.H., Garg S.K., Goland R. (2013). Recommendations for standardizing glucose reporting and analysis to optimize clinical decision making in diabetes: The Ambulatory Glucose Profile (AGP). Diabetes Technol. Ther..

[14] Battelino T., Alexander C.M., Amiel S.A., Arreaza-Rubin G., Beck R.W., Bergenstal R.M., Buckingham B.A., Carroll J., Ceriello A., Chow E. (2023). Continuous glucose monitoring and metrics for clinical trials: An international consensus statement. Lancet Diabetes Endocrinol..

[15] Freckmann G., Eichenlaub M., Waldenmaier D., Pleus S., Wehrstedt S., Haug C., Witthauer L., Jendle J., Hinzmann R., Thomas A. (2023). Clinical Performance Evaluation of Continuous Glucose Monitoring Systems: A Scoping Review and Recommendations for Reporting. J. Diabetes Sci. Technol..

[16] Tricco A.C., Lillie E., Zarin W., O’Brien K.K., Colquhoun H., Levac D., Moher D., Peters M.D.J., Horsley T., Weeks L. (2018). PRISMA Extension for Scoping Reviews (PRISMA-ScR): Checklist and Explanation. Ann. Intern. Med..

[17] OpenAI (2024). ChatGPT (o1) [Large Language Model].

[18] Giray L. (2023). Prompt Engineering with ChatGPT: A Guide for Academic Writers. Ann. Biomed. Eng..

[19] Freedman D., Diaconis P. (1981). On the histogram as a density estimator:L_2_ theory. Z. Wahrscheinlichkeitstheor. Verw. Geb..

[20] OpenAI (2026). ChatGPT (GPT-5.4) [Large Language Model].

[21] Ajčević M., Candido R., Assaloni R., Accardo A., Francescato M.P. (2021). Personalized Approach for the Management of Exercise-Related Glycemic Imbalances in Type 1 Diabetes: Comparison with Reference Method. J. Diabetes Sci. Technol..

[22] Al Ozairi E., ElSamad A., Al Kandari J., Hamdan Y., Taliping D., Gray S.R. (2023). The effect of timing of remotely supervised exercise on glucose control in people with type 1 diabetes during Ramadan: A randomised crossover study. Diabetes Metab. Syndr..

[23] Andersen M.B., Ovesen P.G., Daugaard M., Ostenfeld E.B., Fuglsang J. (2020). Cycling reduces blood glucose excursions after an oral glucose tolerance test in pregnant women: A randomized crossover trial. Appl. Physiol. Nutr. Metab..

[24] Aronson R., Li A., Brown R.E., McGaugh S., Riddell M.C. (2020). Flexible insulin therapy with a hybrid regimen of insulin degludec and continuous subcutaneous insulin infusion with pump suspension before exercise in physically active adults with type 1 diabetes (FIT Untethered): A single-centre, open-label, proof-of-concept, randomised crossover trial. Lancet Diabetes Endocrinol..

[25] Babir F.J., Riddell M.C., Adamo L.M., Richards D.L., Gibala M.J. (2023). The effect of bodyweight exercise on 24-h glycemic responses determined by continuous glucose monitoring in healthy inactive adults: A randomized crossover study. Sci. Rep..

[26] Bauhaus H., Erdogan P., Braun H., Thevis M. (2023). Continuous Glucose Monitoring (CGM) in Sports—A Comparison between a CGM Device and Lab-Based Glucose Analyser under Resting and Exercising Conditions in Athletes. Int. J. Environ. Res. Public Health.

[27] Bowler A.M., Burke L.M., Coffey V.G., Cox G.R. (2025). Day-to-Day Glycemic Variability Using Continuous Glucose Monitors in Endurance Athletes. J. Diabetes Sci. Technol..

[28] Büsing F., Hägele F.A., Nas A., Hasler M., Müller M.J., Bosy-Westphal A. (2019). Impact of energy turnover on the regulation of glucose homeostasis in healthy subjects. Nutr. Diabetes.

[29] Campbell M.D., Alobaid A.M., Hopkins M., Dempsey P.C., Pearson S.M., Kietsiriroje N., Churm R., Ajjan R.A. (2023). Interrupting prolonged sitting with frequent short bouts of light-intensity activity in people with type 1 diabetes improves glycaemic control without increasing hypoglycaemia: The SIT-LESS randomised controlled trial. Diabetes Obes. Metab..

[30] Carter S., Solomon T.P.J. (2020). Exercise-Induced Improvements in Postprandial Glucose Response Are Blunted by Pre-Exercise Hyperglycemia: A Randomized Crossover Trial in Healthy Individuals. Front. Endocrinol..

[31] Chen K., Wang Y., Li D., Li J., Huang Y., Huang M., Ma H. (2024). Impact of diverse aerobic exercise plans on glycemic control, lipid levels, and functional activity in stroke patients with type 2 diabetes mellitus. Front. Endocrinol..

[32] Christiansen M., Bartee A., Lalonde A., Jones R.E., Katz M., Wolpert H., Brazg R. (2021). Performance of an Automated Insulin Delivery System: Results of Early Phase Feasibility Studies. Diabetes Technol. Ther..

[33] Clavel P., Tiollier E., Leduc C., Fabre M., Lacome M., Buchheit M. (2022). Concurrent Validity of a Continuous Glucose-Monitoring System at Rest and During and Following a High-Intensity Interval Training Session. Int. J. Sports Physiol. Perform..

[34] Coates A.M., Cohen J.N., Burr J.F. (2023). Investigating sensor location on the effectiveness of continuous glucose monitoring during exercise in a non-diabetic population. Eur. J. Sport Sci..

[35] Coates A.M., Thompson K.M.A., Grigore M.M., Baker R.E., Pignanelli C., Robertson A.A., Frangos S.M., Cheung C.P., Burr J.F. (2024). Altered carbohydrate oxidation during exercise in overreached endurance athletes is applicable to training monitoring with continuous glucose monitors. Scand. J. Med. Sci. Sports.

[36] Codella R., Gallo G., Meloni A., Luzi L., Filipas L. (2024). Elite Cyclists with Type 1 Diabetes Show Acceptable Glycemic Excursions During a Time-Trial Performance Under High-Definition Transcranial Direct Current Stimulation. Endocr. Pract. Off. J. Am. Coll. Endocrinol. Am. Assoc. Clin. Endocrinol..

[37] Correia I.R., Magalhães J.P., Júdice P.B., Hetherington-Rauth M., Freitas S.P., Lopes J.M., Gama F.F., Sardinha L.B. (2022). Breaking-Up Sedentary Behavior and Detraining Effects on Glycemic Control: A Randomized Crossover Trial in Trained Older Adults. J. Aging Phys. Act..

[38] Cruz L.C.D., Teixeira-Araujo A.A., Passos Andrade K.T., Rocha T.C.O.G., Puga G.M., Moreira S.R. (2019). Low-Intensity Resistance Exercise Reduces Hyperglycemia and Enhances Glucose Control Over a 24-Hour Period in Women With Type 2 Diabetes. J. Strength Cond. Res..

[39] Cuerda Del Pino A., Martín-San Agustín R., José Laguna Sanz A., Díez J.L., Palanca A., Rossetti P., Gumbau-Gimenez M., Ampudia-Blasco F.J., Bondia J. (2024). Accuracy of Two Continuous Glucose Monitoring Devices During Aerobic and High-Intensity Interval Training in Individuals with Type 1 Diabetes. Diabetes Technol. Ther..

[40] Cutruzzolà A., Greco F., Parise M., Irace C., Gnasso A., Emerenziani G.P. (2024). Yoga as an alternative to cycling in type 1 diabetes: A preliminary study of acute effects on glucose levels. J. Sci. Med. Sport.

[41] Da Prato G., Pasquini S., Rinaldi E., Lucianer T., Donà S., Santi L., Negri C., Bonora E., Moghetti P., Trombetta M. (2022). Accuracy of CGM Systems During Continuous and Interval Exercise in Adults with Type 1 Diabetes. J. Diabetes Sci. Technol..

[42] Dole A., Sims S., Gan H., Gill N., Beaven M. (2024). Continuous Glucose Monitoring Underreports Blood Glucose During a Simulated Ultraendurance Run in Eumenorrheic Female Runners. Int. J. Sports Physiol. Perform..

[43] Drenthen L.C.A., Ajie M., Abbink E.J., Rodwell L., Thijssen D.H.J., Tack C.J., de Galan B.E. (2023). No insulin degludec dose adjustment required after aerobic exercise for people with type 1 diabetes: The ADREM study. Diabetologia.

[44] Elghobashy M.E., Richards A.J., Malekzadeh R., Patel D., Turner L.V., Burr J.F., Power G.A., Laham R., Riddell M.C., Cheng A.J. (2024). Carbohydrate Ingestion Increases Interstitial Glucose and Mitigates Neuromuscular Fatigue during Single-Leg Knee Extensions. Med. Sci. Sports Exerc..

[45] Estafanos S., Friesen B., Govette A., Gillen J.B. (2022). Carbohydrate-Energy Replacement Following High-Intensity Interval Exercise Blunts Next-Day Glycemic Control in Untrained Women. Front. Nutr..

[46] Fabris C., Nass R.M., Pinnata J., Carr K.A., Koravi C.L.K., Barnett C.L., Oliveri M.C., Anderson S.M., Chernavvsky D.R., Breton M.D. (2020). The Use of a Smart Bolus Calculator Informed by Real-time Insulin Sensitivity Assessments Reduces Postprandial Hypoglycemia Following an Aerobic Exercise Session in Individuals With Type 1 Diabetes. Diabetes Care.

[47] Figueira F.R., Umpierre D., Bock P.M., Waclawovsky G., Guerra A.P., Donelli A., Andrades M., Casali K.R., Schaan B.D. (2019). Effect of exercise on glucose variability in healthy subjects: Randomized crossover trial. Biol. Sport.

[48] Fujihira K., Takahashi M., Shimamura K., Hayashi N. (2022). Effects of different temperatures of carbohydrate-protein-containing drinks on gastric emptying rate after exercise in healthy young men: Randomized crossover trial. J. Physiol. Anthropol..

[49] Gale J.T., Haszard J.J., Peddie M.C. (2024). Improved glycaemic control induced by evening activity breaks does not persist overnight amongst healthy adults: A randomized crossover trial. Diabetes Obes. Metab..

[50] Gao Y., Li Q.Y., Finni T., Pesola A.J. (2024). Enhanced muscle activity during interrupted sitting improves glycemic control in overweight and obese men. Scand. J. Med. Sci. Sports.

[51] Garcia-Tirado J., Brown S.A., Laichuthai N., Colmegna P., Koravi C.L.K., Ozaslan B., Corbett J.P., Barnett C.L., Pajewski M., Oliveri M.C. (2021). Anticipation of Historical Exercise Patterns by a Novel Artificial Pancreas System Reduces Hypoglycemia During and After Moderate-Intensity Physical Activity in People with Type 1 Diabetes. Diabetes Technol. Ther..

[52] Gómez A.M., Henao D.C., Muñoz O.M., Romero D.M., León J.D.S., Jaramillo P.E., Moscoso E., Parra Prieto D.A., Robledo S., Jaramillo M.G. (2024). Temporary Target Versus Suspended Insulin Infusion in Patients with Type 1 Diabetes Using the MiniMed 780G Advanced Closed-Loop Hybrid System During Aerobic Exercise: A Randomized Crossover Clinical Trial. Diabetes Technol. Ther..

[53] Hall R.M., Dyhrberg S., McTavish A., McTavish L., Corley B., Krebs J.D. (2022). Where can you wear your Libre? Using the FreeStyle Libre continuous glucose monitor on alternative sites. Diabetes Obes. Metab..

[54] Hanaire H., Franc S., Borot S., Penfornis A., Benhamou P.Y., Schaepelynck P., Renard E., Guerci B., Jeandidier N., Simon C. (2020). Efficacy of the Diabeloop closed-loop system to improve glycaemic control in patients with type 1 diabetes exposed to gastronomic dinners or to sustained physical exercise. Diabetes Obes. Metab..

[55] Hásková A., Radovnická L., Petruželková L., Parkin C.G., Grunberger G., Horová E., Navrátilová V., Kádě O., Matoulek M., Prázný M. (2020). Real-time CGM Is Superior to Flash Glucose Monitoring for Glucose Control in Type 1 Diabetes: The CORRIDA Randomized Controlled Trial. Diabetes Care.

[56] Hatamoto Y., Yoshimura E., Takae R., Komiyama T., Matsumoto M., Higaki Y., Tanaka H. (2021). The effects of breaking sedentary time with different intensity exercise bouts on energy metabolism: A randomized cross-over controlled trial. Nutr. Metab. Cardiovasc. Dis. NMCD.

[57] Hiromatsu C., Kasahara N., Lin C.A., Wang F., Goto K. (2023). Continuous Monitoring of Interstitial Fluid Glucose Responses to Endurance Exercise with Different Levels of Carbohydrate Intake. Nutrients.

[58] Holzer R., Schulte-Körne B., Seidler J., Predel H.G., Brinkmann C. (2021). Effects of Acute Resistance Exercise with and without Whole-Body Electromyostimulation and Endurance Exercise on the Postprandial Glucose Regulation in Patients with Type 2 Diabetes Mellitus: A Randomized Crossover Study. Nutrients.

[59] Iida Y., Takeishi S., Fushimi N., Tanaka K., Mori A., Sato Y. (2020). Effect of postprandial moderate-intensity walking for 15-min on glucose homeostasis in type 2 diabetes mellitus patients. Diabetol. Int..

[60] Kim H.K., Furuhashi S., Takahashi M., Chijiki H., Nanba T., Inami T., Radak Z., Sakamoto S., Shibata S. (2022). Late-afternoon endurance exercise is more effective than morning endurance exercise at improving 24-h glucose and blood lipid levels. Front. Endocrinol..

[61] Kleinloog J.P.D., Mensink R.P., Roodt J.O., Thijssen D.H.J., Hesselink M.K.C., Joris P.J. (2022). Aerobic exercise training improves not only brachial artery flow-mediated vasodilatation but also carotid artery reactivity: A randomized controlled, cross-over trial in older men. Physiol. Rep..

[62] Larsen R., Taylor F., Dempsey P.C., McNarry M., Rickards K., Sethi P., Homer A., Cohen N., Owen N., Kumareswaran K. (2025). Effect of Interrupting Prolonged Sitting with Frequent Activity Breaks on Postprandial Glycemia and Insulin Sensitivity in Adults with Type 1 Diabetes on Continuous Subcutaneous Insulin Infusion Therapy: A Randomized Crossover Pilot Trial. Diabetes Technol. Ther..

[63] Lee M.H., Vogrin S., Paldus B., Jayawardene D., Jones H.M., McAuley S.A., Obeyesekere V., Gooley J., La Gerche A., MacIsaac R.J. (2020). Glucose and Counterregulatory Responses to Exercise in Adults With Type 1 Diabetes and Impaired Awareness of Hypoglycemia Using Closed-Loop Insulin Delivery: A Randomized Crossover Study. Diabetes Care.

[64] Lee A.S., Way K.L., Johnson N.A., Twigg S.M. (2020). High-intensity interval exercise and hypoglycaemia minimisation in adults with type 1 diabetes: A randomised cross-over trial. J. Diabetes Its Complicat..

[65] Liu D., Zhang Y., Wu Q., Han R., Cheng D., Wu L., Guo J., Yu X., Ge W., Ni J. (2024). Exercise-induced improvement of glycemic fluctuation and its relationship with fat and muscle distribution in type 2 diabetes. J. Diabetes.

[66] Lu J.C., Morrison D., Halim B., Manos G., Obeyesekere V., Kannard B., Shah R., Wolfe K., Morrow B., Pagliuso B. (2026). Accuracy and Feasibility of a Novel Glucose/Lactate Continuous Multi-Analyte Sensing Platform in Humans. J. Diabetes Sci. Technol..

[67] Lundemose S.B., Laugesen C., Ranjan A.G., Nørgaard K. (2023). Factory-Calibrated Continuous Glucose Monitoring Systems in Type 1 Diabetes: Accuracy during In-Clinic Exercise and Home Use. Sensors.

[68] Macedo A.C.P., Bock P.M., Saffi M.A.L., Madalosso M.M., Lago P.D., Casali K.R., Schaan B.D. (2024). Neuromuscular electrical stimulation changes glucose, but not its variability in type 2 diabetes: A randomized clinical trial. An. Acad. Bras. Cienc..

[69] Marcotte-Chénard A., Tremblay R., Deslauriers L., Geraldes P., Gayda M., Christou D., Mampuya W., Little J.P., Riesco E. (2024). Comparison of 10 × 1-minute high-intensity interval training (HIIT) versus 4 × 4-minute HIIT on glucose control and variability in females with type 2 diabetes. Appl. Physiol. Nutr. Metab..

[70] Mason L.J., Hartwig T., Greene D. (2024). Validating the Use of Continuous Glucose Monitors With Nondiabetic Recreational Runners. Int. J. Sports Physiol. Perform..

[71] Mattsson S., Jendle J., Adolfsson P. (2019). Carbohydrate Loading Followed by High Carbohydrate Intake During Prolonged Physical Exercise and Its Impact on Glucose Control in Individuals with Diabetes Type 1-An Exploratory Study. Front. Endocrinol..

[72] Matzka M., Ørtenblad N., Lenk M., Sperlich B. (2024). Accuracy of a continuous glucose monitoring system applied before, during, and after an intense leg-squat session with low- and high-carbohydrate availability in young adults without diabetes. Eur. J. Appl. Physiol..

[73] McCarthy D.G., Bone J., Fong M., Pinckaers P.J.M., Bostad W., Richards D.L., van Loon L.J.C., Gibala M.J. (2023). Acute Ketone Monoester Supplementation Impairs 20-min Time-Trial Performance in Trained Cyclists: A Randomized, Crossover Trial. Int. J. Sport Nutr. Exerc. Metab..

[74] McClure R.D., Carr A.L.J., Boulé N.G., Yardley J.E. (2024). An Aerobic Cooldown After Morning, Fasted Resistance Exercise Has Limited Impact on Post-exercise Hyperglycemia in Adults With Type 1 Diabetes: A Randomized Crossover Study. Can. J. Diabetes.

[75] Mesa A., Beneyto A., Martín-SanJosé J.F., Viaplana J., Bondia J., Vehí J., Conget I., Giménez M. (2023). Safety and performance of a hybrid closed-loop insulin delivery system with carbohydrate suggestion in adults with type 1 diabetes prone to hypoglycemia. Diabetes Res. Clin. Pract..

[76] Meuffels F.M., Kempe H.P., Becker U., Kornmann M., Kress S., Kreutz T., Brinkmann C. (2023). From Zero to Hero: Type 2 Diabetes Mellitus Patients Hike on the Way of St. James-A Feasibility Study with Analyses of Patients’ Quality of Life, Diabetes Distress and Glucose Profile. Int. J. Environ. Res. Public Health.

[77] Moholdt T., Parr E.B., Devlin B.L., Debik J., Giskeødegård G., Hawley J.A. (2021). The effect of morning vs evening exercise training on glycaemic control and serum metabolites in overweight/obese men: A randomised trial. Diabetologia.

[78] Morrison D., Zaharieva D.P., Lee M.H., Paldus B., Vogrin S., Grosman B., Roy A., Kurtz N., O’Neal D.N. (2022). Comparable Glucose Control with Fast-Acting Insulin Aspart Versus Insulin Aspart Using a Second-Generation Hybrid Closed-Loop System During Exercise. Diabetes Technol. Ther..

[79] Moser O., Pandis M., Aberer F., Kojzar H., Hochfellner D., Elsayed H., Motschnig M., Augustin T., Kreuzer P., Pieber T.R. (2019). A head-to-head comparison of personal and professional continuous glucose monitoring systems in people with type 1 diabetes: Hypoglycaemia remains the weak spot. Diabetes Obes. Metab..

[80] Moser O., Müller A., Aberer F., Aziz F., Kojzar H., Sourij C., Obermayer A., Abbas F., Birnbaumer P., Lenz J. (2023). Comparison of Insulin Glargine 300 U/mL and Insulin Degludec 100 U/mL Around Spontaneous Exercise Sessions in Adults with Type 1 Diabetes: A Randomized Cross-Over Trial (ULTRAFLEXI-1 Study). Diabetes Technol. Ther..

[81] Munan M., Dyck R.A., Houlder S., Yardley J.E., Prado C.M., Snydmiller G., Boulé N.G. (2020). Does Exercise Timing Affect 24-Hour Glucose Concentrations in Adults With Type 2 Diabetes? A Follow Up to the Exercise-Physical Activity and Diabetes Glucose Monitoring Study. Can. J. Diabetes.

[82] Murillo S., Brugnara L., Servitja J.M., Novials A. (2022). High Intensity Interval Training reduces hypoglycemic events compared with continuous aerobic training in individuals with type 1 diabetes: HIIT and hypoglycemia in type 1 diabetes. Diabetes Metab..

[83] Ortega J.F., Morales-Palomo F., Ramirez-Jimenez M., Moreno-Cabañas A., Mora-Rodríguez R. (2020). Exercise improves metformin 72-h glucose control by reducing the frequency of hyperglycemic peaks. Acta Diabetol..

[84] Ozaslan B., Brown S.A., Pinnata J., Barnett C.L., Carr K., Wakeman C.A., Clancy-Oliveri M., Breton M.D. (2022). Safety and Feasibility Evaluation of Step Count Informed Meal Boluses in Type 1 Diabetes: A Pilot Study. J. Diabetes Sci. Technol..

[85] Paing A.C., McMillan K.A., Kirk A.F., Collier A., Hewitt A., Chastin S.F.M. (2019). Dose-response between frequency of breaks in sedentary time and glucose control in type 2 diabetes: A proof of concept study. J. Sci. Med. Sport.

[86] Paldus B., Morrison D., Zaharieva D.P., Lee M.H., Jones H., Obeyesekere V., Lu J., Vogrin S., La Gerche A., McAuley S.A. (2022). A Randomized Crossover Trial Comparing Glucose Control During Moderate-Intensity, High-Intensity, and Resistance Exercise With Hybrid Closed-Loop Insulin Delivery While Profiling Potential Additional Signals in Adults With Type 1 Diabetes. Diabetes Care.

[87] Podestá D.I., Blannin A.K., Wallis G.A. (2024). Effects of overnight-fasted versus fed-state exercise on the components of energy balance and interstitial glucose across four days in healthy adults. Appetite.

[88] Qi Y.Y., Zheng X., Bi L.N., Hu S., Li C., Zhang Y., Shi W.L., Yue Y.J., Li Q. (2025). Effects of postprandial exercise timing on blood glucose and fluctuations in patients with type 2 diabetes mellitus. J. Sports Med. Phys. Fit..

[89] Rafiei H., Robinson E., Barry J., Jung M.E., Little J.P. (2019). Short-term exercise training reduces glycaemic variability and lowers circulating endothelial microparticles in overweight and obese women at elevated risk of type 2 diabetes. Eur. J. Sport Sci..

[90] Raman A., Peiffer J.J., Hoyne G.F., Lawler N.G., Currie A., Fairchild T.J. (2023). Exercise-induced responses in matrix metalloproteinases and osteopontin are not moderated by exercise format in males with overweight or obesity. Eur. J. Appl. Physiol..

[91] Rees J.L., Chang C.R., François M.E., Marcotte-Chénard A., Fontvieille A., Klaprat N.D., Dyck R.A., Funk D.R., Snydmiller G., Bastell K. (2019). Minimal effect of walking before dinner on glycemic responses in type 2 diabetes: Outcomes from the multi-site E-PAraDiGM study. Acta Diabetol..

[92] Reid L.A., Rees J.L., Kimber M., James M., Purdy G.M., Smorschok M., Maier L.E., Boulé N.G., Day T.A., Davenport M.H. (2025). Blood Glucose During High Altitude Trekking in Young Healthy Adults. High Alt. Med. Biol..

[93] Savikj M., Gabriel B.M., Alm P.S., Smith J., Caidahl K., Björnholm M., Fritz T., Krook A., Zierath J.R., Wallberg-Henriksson H. (2019). Afternoon exercise is more efficacious than morning exercise at improving blood glucose levels in individuals with type 2 diabetes: A randomised crossover trial. Diabetologia.

[94] Schein A.S.O., Corrêa A.P.S., Macedo A.C.P., Dartora D.R., da Silveira A.D., Severo M.D., Casali K.R., Schaan B.D. (2020). Acute inspiratory muscle exercise effect on glucose levels, glucose variability and autonomic control in patients with type 2 diabetes: A crossover randomized trial. Auton. Neurosci. Basic Clin..

[95] Schleh M.W., Pitchford L.M., Gillen J.B., Horowitz J.F. (2020). Energy Deficit Required for Exercise-induced Improvements in Glycemia the Next Day. Med. Sci. Sports Exerc..

[96] Scott S.N., Cocks M., Andrews R.C., Narendran P., Purewal T.S., Cuthbertson D.J., Wagenmakers A.J.M., Shepherd S.O. (2019). Fasted High-Intensity Interval and Moderate-Intensity Exercise Do Not Lead to Detrimental 24-Hour Blood Glucose Profiles. J. Clin. Endocrinol. Metab..

[97] Scott S.N., Cocks M., Andrews R.C., Narendran P., Purewal T.S., Cuthbertson D.J., Wagenmakers A.J.M., Shepherd S.O. (2019). High-Intensity Interval Training Improves Aerobic Capacity Without a Detrimental Decline in Blood Glucose in People With Type 1 Diabetes. J. Clin. Endocrinol. Metab..

[98] Sevilla-Lorente R., Marmol-Perez A., Gonzalez-Garcia P., Rodríguez-Miranda M.L.N., Riquelme-Gallego B., Aragon-Vela J., Martinez-Gálvez J.M., Molina-Garcia P., Alcantara J.M.A., Garcia-Consuegra J. (2025). Sexual dimorphism on the acute effect of exercise in the morning vs. evening: A randomized crossover study. J. Sport Health Sci..

[99] Shambrook P., Kingsley M.I., Taylor N.F., Wundersitz D.W., Wundersitz C.E., Paton C.D., Gordon B.A. (2020). A comparison of acute glycaemic responses to accumulated or single bout walking exercise in apparently healthy, insufficiently active adults. J. Sci. Med. Sport.

[100] Solomon T.P.J., Tarry E., Hudson C.O., Fitt A.I., Laye M.J. (2020). Immediate post-breakfast physical activity improves interstitial postprandial glycemia: A comparison of different activity-meal timings. Pflug. Arch. Eur. J. Physiol..

[101] Sparks J.R., Sarzynski M.A., Davis J.M., Grandjean P.W., Wang X. (2021). Alterations in Glycemic Variability, Vascular Health, and Oxidative Stress following a 12-Week Aerobic Exercise Intervention-A Pilot Study. Int. J. Exerc. Sci..

[102] Tanaka Y., Ogata H., Park I., Ando A., Ishihara A., Kayaba M., Yajima K., Suzuki C., Araki A., Osumi H. (2021). Effect of a single bout of morning or afternoon exercise on glucose fluctuation in young healthy men. Physiol. Rep..

[103] Tee C.C.L., Parr E.B., Cooke M.B., Chong M.C., Rahmat N., Md Razali M.R., Yeo W.K., Camera D.M. (2023). Combined effects of exercise and different levels of acute hypoxic severity: A randomized crossover study on glucose regulation in adults with overweight. Front. Physiol..

[104] Tee C.C.L., Chong M.C., Cooke M.B., Rahmat N., Yeo W.K., Camera D.M. (2024). Effects of exercise modality combined with moderate hypoxia on blood glucose regulation in adults with overweight. Front. Physiol..

[105] Toghi-Eshghi S.R., Yardley J.E. (2019). Morning (Fasting) vs Afternoon Resistance Exercise in Individuals With Type 1 Diabetes: A Randomized Crossover Study. J. Clin. Endocrinol. Metab..

[106] van Meijel R.L.J., Blaak E.E., Goossens G.H. (2023). Effects of hypoxic exercise on 24-hour glucose profile and substrate metabolism in overweight and obese men with impaired glucose metabolism. Am. J. Physiol. Endocrinol. Metab..

[107] Wowdzia J.B., Hazell T.J., Davenport M.H. (2022). Glycemic response to acute high-intensity interval versus moderate-intensity continuous exercise during pregnancy. Physiol. Rep..

[108] Yardley J.E., Rees J.L., Funk D.R., Toghi-Eshghi S.R., Boulé N.G., Senior P.A. (2019). Effects of Moderate Cycling Exercise on Blood Glucose Regulation Following Successful Clinical Islet Transplantation. J. Clin. Endocrinol. Metab..

[109] Yardley J.E. (2020). Fasting May Alter Blood Glucose Responses to High-Intensity Interval Exercise in Adults With Type 1 Diabetes: A Randomized, Acute Crossover Study. Can. J. Diabetes.

[110] Zaharieva D.P., Cinar A., Yavelberg L., Jamnik V., Riddell M.C. (2020). No Disadvantage to Insulin Pump Off vs Pump On During Intermittent High-Intensity Exercise in Adults With Type 1 Diabetes. Can. J. Diabetes.

[111] Zhang X., Wongpipit W., Sun F., Sheridan S., Huang W.Y.J., Sit C.H.P., Wong S.H.S. (2021). Walking Initiated 20 Minutes before the Time of Individual Postprandial Glucose Peak Reduces the Glucose Response in Young Men with Overweight or Obesity: A Randomized Crossover Study. J. Nutr..

[112] Zhang X., Tian X.Y., Miyashita M., Sun F., Huang W.Y.J., Zheng C., Sum M.K., Wong S.H.S. (2023). Effects of accumulated versus continuous individualized exercise on postprandial glycemia in young adults with obesity. Eur. J. Sport Sci..

[113] Zheng X., Qi Y., Bi L., Shi W., Zhang Y., Zhao D., Hu S., Li M., Li Q. (2020). Effects of Exercise on Blood Glucose and Glycemic Variability in Type 2 Diabetic Patients with Dawn Phenomenon. BioMed Res. Int..

[114] Li A., Riddell M.C., Potashner D., Brown R.E., Aronson R. (2019). Time Lag and Accuracy of Continuous Glucose Monitoring During High Intensity Interval Training in Adults with Type 1 Diabetes. Diabetes Technol. Ther..

[115] Maytham K., Hagelqvist P.G., Engberg S., Forman J.L., Pedersen-Bjergaard U., Knop F.K., Vilsbøll T., Andersen A. (2024). Accuracy of continuous glucose monitoring during exercise-related hypoglycemia in individuals with type 1 diabetes. Front. Endocrinol..

[116] Zaharieva D.P., Turksoy K., McGaugh S.M., Pooni R., Vienneau T., Ly T., Riddell M.C. (2019). Lag Time Remains with Newer Real-Time Continuous Glucose Monitoring Technology During Aerobic Exercise in Adults Living with Type 1 Diabetes. Diabetes Technol. Ther..

[117] Moser O., Riddell M.C., Eckstein M.L., Adolfsson P., Rabasa-Lhoret R., van den Boom L., Gillard P., Nørgaard K., Oliver N.S., Zaharieva D.P. (2020). Glucose management for exercise using continuous glucose monitoring (CGM) and intermittently scanned CGM (isCGM) systems in type 1 diabetes: Position statement of the European Association for the Study of Diabetes (EASD) and of the International Society for Pediatric and Adolescent Diabetes (ISPAD) endorsed by JDRF and supported by the American Diabetes Association (ADA). Diabetologia.

[118] Bowler A.M., Whitfield J., Marshall L., Coffey V.G., Burke L.M., Cox G.R. (2022). The Use of Continuous Glucose Monitors in Sport: Possible Applications and Considerations. Int. J. Sport Nutr. Exerc. Metab..

[119] Englert K., Ruedy K., Coffey J., Caswell K., Steffen A., Levandoski L. (2014). Diabetes Research in Children (DirecNet) Study Group. Skin and adhesive issues with continuous glucose monitors: A sticky situation. J. Diabetes Sci. Technol..

[120] Francois M.E., Cosgrove S.D., Walker N.M., Lucas S.J., Black K.E. (2018). Physiological responses to a five-day adventure race: Continuous blood glucose, hemodynamics and metabolites the 2012 GODZone field-study. J. Exerc. Sci. Fit..

[121] (2013). In Vitro Diagnostic Test Systems—Requirements for Blood-Glucose Monitoring Systems for Self-Testing in Managing Diabetes Mellitus.

