# Supplementary material for: Use of Continuous Glucose Monitors in Exercise Research Studies—A Scoping Review on Study Characteristics and Common Practices
Source: Sports (Basel). 2026 Jul 2;14(7):274. doi: 10.3390/sports14070274 (PMC13417461; doi:10.3390/sports14070274)
Supplement: Supplementary file 1 [file sports-14-00274-s001.zip › sports-4359211-supplementary File S1-S3.pdf]

Supplementary Materials

Supplemental Material S1: Prompt

Prompt Used for Eligibility Screening

Instruction
You are assisting with a systematic review titled "Use of continuous glucose monitors in exercise research studies – A systematic analysis of study characteristics and common practices." Your task is to determine whether the given publication meets **all** the inclusion criteria, fails **one or more** criteria (leading to exclusion), or if the information provided is **insufficient** to make a clear decision.

Context

- **Inclusion Criteria**:
    1. The study includes an **exercise intervention** (acute or chronic) with **supervised, structured exercise**, providing detailed information on volume, frequency and intensity.
        - Permissible combinations:
            - Structured, supervised training **plus** non-supervised training (e.g. home training).
            - Exercise **plus** nutritional and/or medication intervention.
        - Inadmissible designs:
            - **Purely non-supervised** training (home-based, only monitored by sensors or trackers, training recommendations given solely as educational or motivational interventions, or training videos for unsupervised use).
    2. The study employs **continuous glucose monitoring (CGM) during or after** the exercise intervention for analysis.
    3. The study population includes **adults (≥18 years old)**, whether healthy or with a given condition (e.g. T1DM, T2DM, obesity, athletes, etc.).
    4. The publication is **original research** (not a review, meta-analysis, observational study, or pure study protocol).
- **Exclusion**: If the text indicates that **one or more** of these criteria are not met, the publication is excluded.
- **Uncertainty**: If the text **does not provide enough information** to determine inclusion or exclusion, answer **"Maybe"**.

Input Data
[Provide here the abstract or relevant summary of the publication that needs to be evaluated.]

Output Indicator
Please output **one single word—"Include"**, **"Exclude"**, or **"Maybe"**—without any additional explanation or commentary.

Supplemental Material S2: Prompt

Prompt Used for Data Extraction

(1) Role & Task
You are an information extraction model. From the attached PDF of a scientific article about Continuous Glucose Monitoring (CGM), extract ONLY information that is explicitly stated in the article text (no inference). When something is not explicitly stated, return "Not stated."

(2) What to extract (with controlled vocab)
Return the following fields. Use the exact category labels below where categories are specified.

Study design — choose exactly one of:
• randomized crossover design
• randomized parallel-group design
• single-group non-crossover design
• non-randomized crossover design
• non-randomized parallel-group design

Study type — choose exactly one of:
• Validation study (primary goal: analyze the accuracy of CGM devices)
• Non-validation study (primary goal: analyze glucose dynamics in various situations)

Study duration (single field; acute vs chronic) — choose exactly one of:
• Acute exercise exposure
• Chronic exercise exposure
(Use the rules in Section 5. If information required by the rules is not explicitly reported, return "Not stated.")

Number of subjects — per rules in Section 5 (report **one** number).

Type of subjects — choose any that apply (multiple allowed):
• subjects with T1DM
• subjects with T2DM
• subjects with other forms of diabetes (e.g., gestational diabetes)
• non-diabetic subjects (incl. healthy, overweight, obese)
• athletes (only if explicitly stated)
• other subjects

CGM system (split into two subfields):
• **CGM system — manufacturer/company:** report the explicit manufacturer/company name(s) stated (e.g., "Abbott," "Dexcom," "Medtronic").
• **CGM system — version/variant (as written):** report the explicit model/version wording exactly as stated (e.g., "FreeStyle Libre 2," "Dexcom G6," "Libre Sense"). If unclear or not stated, return "Not stated."

Sensor attachment site — as stated (e.g. "upper arm," "abdomen").

Reported parameters of the CGM system — list all outcome metrics actually reported (e.g., time in range, time <70 mg/dL, MARD, mean glucose, SD, CV, % time >180 mg/dL, AUC, etc.). Include units if stated.

(3) Source & Scope
• Use only the text in the attached PDF.
• Prefer definitive statements from Methods; if conflicting with Abstract, use Methods. If Methods and Results conflict, prefer Methods for design/sample, Results for outcomes.

• If multiple studies/arms/cohorts exist, extract per study and note arms in the citation if relevant.

(4) Citation Requirements
For every field you return, add a citation in parentheses with:
• Section/heading (if present),
• page number (PDF page as displayed), and
• a short quoted fragment (≤15 words) that justifies the value.
Format: **(Methods, p. 5: "randomized crossover")**
If the article uses line/figure labels instead of headings, cite the closest identifiable locator: **(p. 6, para. 2: "upper arm")**.

(5) Disambiguation & Mapping Rules
• Do not infer. If wording does not clearly map to a controlled term, output “Not stated.”
• If more than one category could apply, choose the most specific that is explicitly stated; otherwise “Not stated.”

Number of subjects (apply ALL of the following, in order):

1. Otherwise, **report the analyzed sample size**. If several analyzed Ns are reported for different outcomes/arms/timepoints, **report the largest analyzed N** that pertains to CGM outcomes.
2. If only “enrolled” is given and **no analyzed N** is reported anywhere, return **“Not stated.”**
   (Always cite the exact sentence/table line supporting the reported N.)

Study duration (acute vs chronic) — apply in this order; all inputs must be explicitly reported:

1. **Progression/Program (highest priority):** Classify as Chronic exercise exposure only if a progressive/periodized training plan is reported **and** the longest continuous training block per arm is > 7 calendar days or includes ≥ 8 training sessions.
2. **Dose-only fallback (no progression stated) + author label tie-breaker:** If there is no explicit progression/periodization, classify as Chronic exercise exposure when the longest continuous training block per arm is > 7 calendar days or includes ≥ 8 sessions; otherwise classify as Acute exercise exposure. If authors explicitly state “acute” and the dose is ≤ 7 days and < 8 sessions, classify as Acute exercise exposure. If authors state “training program,” “intervention,” or “adaptations,” still require the minimum dose above before classifying as Chronic training exposure.
3. **Multi-arm/crossover studies:** Classify **each arm** using rules (1)–(2) without counting washout. The **overall study** is **Acute exercise exposure** only if **all arms** are acute; if **any arm** is chronic, classify the study as **Chronic training exposure**.
4. **Monitoring-only periods** (e.g., 14-day CGM without training) **do not** count toward block length.
5. If the article does not explicitly report what is needed for these rules, return **“Not stated.”**

CGM system (manufacturer & version):
• If **multiple CGM systems** are used, **list each** manufacturer and its stated version/variant; if the version for a listed system is not explicitly stated, return **“Not stated”** for that system’s version/variant.
• Do not infer versions from release dates, images, apps, readers, or context.

CGM parameters: include outcome metrics (performance or glycemic metrics), not hardware specs unless the article explicitly reports them as outcomes (e.g. MARD is acceptable; sensor life is not unless analyzed as an outcome).

(6) Output Format
Return a concise bullet list exactly in this order and format:
• **Study design:** [value] (Section/Page + short quote)
• **Study type:** [value] (Section/Page + short quote)
• **Study duration:** [value] (Section/Page + short quote)
• **Number of subjects:** [single number per rules] (Section/Page + short quote)
• **Type of subjects:** [value(s)] (Section/Page + short quote)
• **CGM system — manufacturer/company:** [value(s)] (Section/Page + short quote)
• **CGM system — version/variant (as written):** [value(s) exactly as in text; "Not stated" if unclear] (Section/Page + short quote)
• **Sensor attachment site:** [value] (Section/Page + short quote)
• **Reported parameters of the CGM system:** [comma-separated list with units if stated] (Section/Page + short quote)

Where a field is missing:

- **[Field name]:** Not stated.

(7) Quality Checks (silent, no extra output)
• Verify each non–"Not stated" field has a valid category (where applicable) and a citation as specified.
• If the article has multiple studies, repeat the bullet list per study with a short study label (e.g. "Study A," "Study B") taken from the paper.

Supplemental Material S3: Table

Table of Publications Included in the Analyses (93 studies)

First author, year	Full Title	Journal	DOI/PMID
Ajčević et al., 2021	Personalized Approach for the Management of Exercise-Related Glycemic Imbalances in Type 1 Diabetes: Comparison with Reference Method	Journal of Diabetes Science and Technology	https://doi.org/10.1177/1932296820945372
Al Ozairi et al., 2023	The effect of timing of remotely supervised exercise on glucose control in people with type 1 diabetes during Ramadan: A randomised crossover study	Diabetes & Metabolic Syndrome	https://doi.org/10.1016/j.dsx.2023.102845
Andersen et al., 2020	Cycling reduces blood glucose excursions after an oral glucose tolerance test in pregnant women: a randomized crossover trial	Applied Physiology, Nutrition, and Metabolism	https://doi.org/10.1139/apnm-2020-0020
Aronson et al., 2020	Flexible insulin therapy with a hybrid regimen of insulin degludec and continuous subcutaneous insulin infusion with pump suspension before exercise in physically active adults with type 1 diabetes (FIT Untethered): a single-centre, open-label, proof-of-concept, randomised crossover trial	The Lancet Diabetes & Endocrinology	https://doi.org/10.1016/S2213-8587(20)30114-5
Babir et al., 2023	The effect of bodyweight exercise on 24-h glycemic responses determined by continuous glucose monitoring in healthy inactive adults: a randomized crossover study	Scientific Reports	https://doi.org/10.1038/s41598-023-48063-y
Bauhaus et al., 2023	Continuous Glucose Monitoring (CGM) in Sports—A Comparison between a CGM Device and Lab-Based Glucose Analyser under Resting and Exercising Conditions in Athletes	International Journal of Environmental Research and Public Health	https://doi.org/10.3390/ijerph20156440
Bowler et al., 2025	Day-to-Day Glycemic Variability Using Continuous Glucose Monitors in Endurance Athletes	Journal of Diabetes Science and Technology	https://doi.org/10.1177/19322968241250355
Büsing et al., 2019	Impact of energy turnover on the regulation of glucose homeostasis in healthy subjects	Nutrition & Diabetes	https://doi.org/10.1038/s41387-019-0089-6
Campbell et al., 2023	Interrupting prolonged sitting with frequent short bouts of light-intensity activity in people with type 1 diabetes improves glycaemic control without increasing hypoglycaemia: The SIT-LESS randomised controlled trial	Diabetes, Obesity and Metabolism	https://doi.org/10.1111/dom.15254
Carter & Solomon, 2020	Exercise-Induced Improvements in Postprandial Glucose Response Are Blunted by Pre-Exercise Hyperglycemia: A Randomized Crossover Trial in Healthy Individuals	Frontiers in Endocrinology	https://doi.org/10.3389/fendo.2020.566548
Chen et al., 2024	Impact of diverse aerobic exercise plans on glycemic control, lipid levels, and functional activity in stroke patients with type 2 diabetes mellitus	Frontiers in Endocrinology	https://doi.org/10.3389/fendo.2024.1389538
Christiansen et al., 2021	Performance of an Automated Insulin Delivery System: Results of Early Phase Feasibility Studies	Diabetes Technology & Therapeutics	https://doi.org/10.1089/dia.2020.0318

Clavel et al., 2022	Concurrent Validity of a Continuous Glucose-Monitoring System at Rest and During and Following a High-Intensity Interval Training Session	International Journal of Sports Physiology and Performance	https://doi.org/10.1123/ijspp.2021-0222
Coates et al., 2023	Investigating sensor location on the effectiveness of continuous glucose monitoring during exercise in a non-diabetic population	European Journal of Sport Science	https://doi.org/10.1080/17461391.2023.2174452
Coates et al., 2024	Altered carbohydrate oxidation during exercise in overreached endurance athletes is applicable to training monitoring with continuous glucose monitors	Scandinavian Journal of Medicine & Science in Sports	https://doi.org/10.1111/sms.14551
Codella et al., 2024	Elite Cyclists with Type 1 Diabetes Show Acceptable Glycemic Excursions During a Time-Trial Performance Under High-Definition Transcranial Direct Current Stimulation	Endocrine Practice:	https://doi.org/10.1016/j.eprac.2024.01.012
Correia et al., 2023	Breaking-Up Sedentary Behavior and Detraining Effects on Glycemic Control: A Randomized Crossover Trial in Trained Older Adults	Journal of Aging and Physical Activity	https://doi.org/10.1123/japa.2022-0124
Cruz et al., 2019	Low-Intensity Resistance Exercise Reduces Hyperglycemia and Enhances Glucose Control Over a 24-Hour Period in Women With Type 2 Diabetes	Journal of Strength and Conditioning Research	https://doi.org/10.1519/JSC.0000000000002410
Cuerda del Pino et al., 2024	Accuracy of Two Continuous Glucose Monitoring Devices During Aerobic and High-Intensity Interval Training in Individuals with Type 1 Diabetes	Diabetes Technology & Therapeutics	https://doi.org/10.1089/dia.2023.0535
Cutruzzolà et al., 2024	Yoga as an alternative to cycling in type 1 diabetes: A preliminary study of acute effects on glucose levels	Journal of Science and Medicine in Sport	https://doi.org/10.1016/j.jsams.2024.06.004
Da Prato et al., 2022	Accuracy of CGM Systems During Continuous and Interval Exercise in Adults with Type 1 Diabetes	Journal of Diabetes Science and Technology	https://doi.org/10.1177/19322968211023522
Dole et al., 2024	Continuous Glucose Monitoring Underreports Blood Glucose During a Simulated Ultraendurance Run in Eumenorrheic Female Runners	International Journal of Sports Physiology and Performance	https://doi.org/10.1123/ijspp.2024-0068
Drenthen et al., 2023	No insulin degludec dose adjustment required after aerobic exercise for people with type 1 diabetes: the ADREM study	Diabetologia	https://doi.org/10.1007/s00125-023-05893-9
Elghobashy et al., 2024	Carbohydrate Ingestion Increases Interstitial Glucose and Mitigates Neuromuscular Fatigue during Single-Leg Knee Extensions	Medicine and Science in Sports and Exercise	https://doi.org/10.1249/MSS.0000000000003440
Estafanos et al., 2022	Carbohydrate-Energy Replacement Following High-Intensity Interval Exercise Blunts Next-Day Glycemic Control in Untrained Women	Frontiers in Nutrition	https://doi.org/10.3389/fnut.2022.868511
Fabris et al., 2020	The Use of a Smart Bolus Calculator Informed by Real-time Insulin Sensitivity Assessments Reduces Postprandial Hypoglycemia Following an Aerobic Exercise Session in Individuals With Type 1 Diabetes	Diabetes Care	https://doi.org/10.2337/dc19-1675

Figueira et al., 2019	Effect of exercise on glucose variability in healthy subjects: randomized crossover trial	Biology of Sport	https://doi.org/10.5114/biolsport.2019.83006
Fujihira et al., 2022	Effects of different temperatures of carbohydrate-protein-containing drinks on gastric emptying rate after exercise in healthy young men: randomized crossover trial	Journal of Physiological Anthropology	https://doi.org/10.1186/s40101-022-00311-2
Gale et al., 2024	Improved glycaemic control induced by evening activity breaks does not persist overnight amongst healthy adults: A randomized crossover trial	Diabetes, Obesity & Metabolism	https://doi.org/10.1111/dom.15589
Gao et al., 2024	Enhanced muscle activity during interrupted sitting improves glycemic control in overweight and obese men	Scandinavian Journal of Medicine & Science in Sports	https://doi.org/10.1111/sms.14628
Garcia-Tirado et al., 2021	Anticipation of Historical Exercise Patterns by a Novel Artificial Pancreas System Reduces Hypoglycemia During and After Moderate-Intensity Physical Activity in People with Type 1 Diabetes	Diabetes Technology & Therapeutics	https://doi.org/10.1089/dia.2020.0516
Gómez et al., 2024	Temporary Target Versus Suspended Insulin Infusion in Patients with Type 1 Diabetes Using the MiniMed 780G Advanced Closed-Loop Hybrid System During Aerobic Exercise: A Randomized Crossover Clinical Trial	Diabetes Technology & Therapeutics	https://doi.org/10.1089/dia.2023.0589
Hall et al., 2022	Where can you wear your Libre? Using the FreeStyle Libre continuous glucose monitor on alternative sites	Diabetes, Obesity and Metabolism	https://doi.org/10.1111/dom.14630
Hanaire et al., 2020	Efficacy of the Diabeloop closed-loop system to improve glycaemic control in patients with type 1 diabetes exposed to gastronomic dinners or to sustained physical exercise	Diabetes, Obesity & Metabolism	https://doi.org/10.1111/dom.13898
Hásková et al., 2020	Real-time CGM Is Superior to Flash Glucose Monitoring for Glucose Control in Type 1 Diabetes: The CORRIDA Randomized Controlled Trial	Diabetes Care	https://doi.org/10.2337/dc20-0112
Hatamoto et al., 2021	The effects of breaking sedentary time with different intensity exercise bouts on energy metabolism: A randomized cross-over controlled trial	Nutrition, Metabolism, and Cardiovascular Diseases	https://doi.org/10.1016/j.numecd.2021.03.006
Hiromatsu et al., 2023	Continuous Monitoring of Interstitial Fluid Glucose Responses to Endurance Exercise with Different Levels of Carbohydrate Intake	Nutrients	https://doi.org/10.3390/nu15224746
Holzer et al., 2021	Effects of Acute Resistance Exercise with and without Whole-Body Electromyostimulation and Endurance Exercise on the Postprandial Glucose Regulation in Patients with Type 2 Diabetes Mellitus: A Randomized Crossover Study	Nutrients	https://doi.org/10.3390/nu13124322
Iida et al., 2020	Effect of postprandial moderate-intensity walking for 15-min on glucose homeostasis in type 2 diabetes mellitus patients	Diabetology International	https://doi.org/10.1007/s13340-020-00433-x
Kim et al., 2022	Late-afternoon endurance exercise is more effective than morning endurance exercise at improving 24-h glucose and blood lipid levels	Frontiers in Endocrinology	https://doi.org/10.3389/fendo.2022.957239

Kleinloog et al., 2022	Aerobic exercise training improves not only brachial artery flow-mediated vasodilatation but also carotid artery reactivity: A randomized controlled, cross-over trial in older men	Physiological Reports	https://doi.org/10.14814/phy2.15395
Larsen et al., 2025	Effect of Interrupting Prolonged Sitting with Frequent Activity Breaks on Postprandial Glycemia and Insulin Sensitivity in Adults with Type 1 Diabetes on Continuous Subcutaneous Insulin Infusion Therapy: A Randomized Crossover Pilot Trial	Diabetes Technology & Therapeutics	https://doi.org/10.1089/dia.2024.0146
Lee, Vogrin et al., 2020	Glucose and Counterregulatory Responses to Exercise in Adults With Type 1 Diabetes and Impaired Awareness of Hypoglycemia Using Closed-Loop Insulin Delivery: A Randomized Crossover Study	Diabetes Care	https://doi.org/10.2337/dc19-1433
Lee, Way et al., 2020	High-intensity interval exercise and hypoglycaemia minimisation in adults with type 1 diabetes: A randomised cross-over trial	Journal of Diabetes and its Complications	https://doi.org/10.1016/j.jdiacomp.2019.107514
Liu et al., 2024	Exercise-induced improvement of glycemic fluctuation and its relationship with fat and muscle distribution in type 2 diabetes	Journal of Diabetes	https://doi.org/10.1111/1753-0407.13549
Lu et al., 2026	Accuracy and Feasibility of a Novel Glucose/Lactate Continuous Multi-Analyte Sensing Platform in Humans	Journal of Diabetes Science and Technology	https://doi.org/10.1177/19322968241266822
Lundemose et al., 2023	Factory-Calibrated Continuous Glucose Monitoring Systems in Type 1 Diabetes: Accuracy during In-Clinic Exercise and Home Use	Sensors	https://doi.org/10.3390/s23229256
Macedo et al., 2024	Neuromuscular electrical stimulation changes glucose, but not its variability in type 2 diabetes: a randomized clinical trial	Anais da Academia Brasileira de Ciências	https://doi.org/10.1590/0001-3765202320220282
Marcotte-Chénard et al., 2024	Comparison of 10 × 1-minute high-intensity interval training (HIIT) versus 4 × 4-minute HIIT on glucose control and variability in females with type 2 diabetes	Applied Physiology, Nutrition, and Metabolism	https://doi.org/10.1139/apnm-2023-0326
Mason et al., 2024	Validating the Use of Continuous Glucose Monitors With Nondiabetic Recreational Runners	International Journal of Sports Physiology and Performance	https://doi.org/10.1123/ijspp.2024-0102
Mattsson et al., 2019	Carbohydrate Loading Followed by High Carbohydrate Intake During Prolonged Physical Exercise and Its Impact on Glucose Control in Individuals With Diabetes Type 1-An Exploratory Study	Frontiers in Endocrinology	https://doi.org/10.3389/fendo.2019.00571
Matzka et al., 2024	Accuracy of a continuous glucose monitoring system applied before, during, and after an intense leg-squat session with low- and high-carbohydrate availability in young adults without diabetes	European Journal of Applied Physiology	https://doi.org/10.1007/s00421-024-05557-5
McCarthy et al., 2023	Acute Ketone Monoester Supplementation Impairs 20-min Time-Trial Performance in Trained	International Journal of Sport Nutrition and	https://doi.org/10.1123/ijsnem.2022-0255

	Cyclists: A Randomized, Crossover Trial	Exercise Metabolism	
McClure et al., 2024	An Aerobic Cooldown After Morning, Fasted Resistance Exercise Has Limited Impact on Post-exercise Hyperglycemia in Adults With Type 1 Diabetes: A Randomized Crossover Study	Canadian Journal of Diabetes	https://doi.org/10.1016/j.jcjd.2024.05.001
Mesa et al., 2023	Safety and performance of a hybrid closed-loop insulin delivery system with carbohydrate suggestion in adults with type 1 diabetes prone to hypoglycemia	Diabetes Research and Clinical Practice	https://doi.org/10.1016/j.diabres.2023.110956
Meuffels et al., 2023	From Zero to Hero: Type 2 Diabetes Mellitus Patients Hike on the Way of St. James—A Feasibility Study with Analyses of Patients' Quality of Life, Diabetes Distress and Glucose Profile	International Journal of Environmental Research and Public Health	https://doi.org/10.3390/ijerph20021417
Moholdt et al., 2021	The effect of morning vs evening exercise training on glycaemic control and serum metabolites in overweight/obese men: a randomised trial	Diabetologia	https://doi.org/10.1007/s00125-021-05477-5
Morrison et al., 2022	Comparable Glucose Control with Fast-Acting Insulin Aspart Versus Insulin Aspart Using a Second-Generation Hybrid Closed-Loop System During Exercise	Diabetes Technology & Therapeutics	https://doi.org/10.1089/dia.2021.0221
Moser et al., 2019	A head-to-head comparison of personal and professional continuous glucose monitoring systems in people with type 1 diabetes: Hypoglycaemia remains the weak spot	Diabetes, Obesity & Metabolism	https://doi.org/10.1111/dom.13598
Moser et al., 2023	Comparison of Insulin Glargine 300 U/mL and Insulin Degludec 100 U/mL Around Spontaneous Exercise Sessions in Adults with Type 1 Diabetes: A Randomized Cross-Over Trial (ULTRAFLEXI-1 Study)	Diabetes Technology & Therapeutics	https://doi.org/10.1089/dia.2022.0422
Munan et al., 2020	Does Exercise Timing Affect 24-Hour Glucose Concentrations in Adults With Type 2 Diabetes? A Follow Up to the Exercise-Physical Activity and Diabetes Glucose Monitoring Study	Canadian Journal of Diabetes	https://doi.org/10.1016/j.jcjd.2020.05.012
Murillo et al., 2022	High Intensity Interval Training reduces hypoglycemic events compared with continuous aerobic training in individuals with type 1 diabetes: HIIT and hypoglycemia in type 1 diabetes	Diabetes & Metabolism	https://doi.org/10.1016/j.diabet.2022.101361
Ortega et al., 2020	Exercise improves metformin 72-h glucose control by reducing the frequency of hyperglycemic peaks	Acta Diabetologia	https://doi.org/10.1007/s00592-020-01488-7
Ozaslan et al., 2022	Safety and Feasibility Evaluation of Step Count Informed Meal Boluses in Type 1 Diabetes: A Pilot Study	Journal of Diabetes Science and Technology	https://doi.org/10.1177/19322968211997917
Paing et al., 2019	Dose-response between frequency of breaks in sedentary time and glucose control in type 2 diabetes: A proof of concept study	Journal of Science and Medicine in Sport	https://doi.org/10.1016/j.jsams.2019.01.017
Paldus et al., 2022	A Randomized Crossover Trial Comparing Glucose Control During Moderate-Intensity, High-Intensity, and Resistance Exercise With Hybrid Closed-Loop Insulin Delivery While	Diabetes Care	https://doi.org/10.2337/dc21-1593

	Profiling Potential Additional Signals in Adults With Type 1 Diabetes		
Podestá et al., 2024	Effects of overnight-fasted versus fed-state exercise on the components of energy balance and interstitial glucose across four days in healthy adults	Appetite	https://doi.org/10.1016/j.appet.2024.107716
Qi et al., 2025	Effects of postprandial exercise timing on blood glucose and fluctuations in patients with type 2 diabetes mellitus	The Journal of Sports Medicine and Physical Fitness	https://doi.org/10.23736/S0022-4707.24.16076-8
Rafiei et al., 2019	Short-term exercise training reduces glycaemic variability and lowers circulating endothelial microparticles in overweight and obese women at elevated risk of type 2 diabetes	European Journal of Sport Science	https://doi.org/10.1080/17461391.2019.1576772
Raman et al., 2023	Exercise-induced responses in matrix metalloproteinases and osteopontin are not moderated by exercise format in males with overweight or obesity	European Journal of Applied Physiology	https://doi.org/10.1007/s00421-023-05133-3
Rees et al., 2019	Minimal effect of walking before dinner on glycemic responses in type 2 diabetes: outcomes from the multi-site E-PAraDiGM study	Acta Diabetologica	https://doi.org/10.1007/s00592-019-01358-x
Reid et al., 2025	Blood Glucose During High Altitude Trekking in Young Healthy Adults	High Altitude Medicine & Biology	https://doi.org/10.1089/ham.2024.0070
Savikj et al., 2019	Afternoon exercise is more efficacious than morning exercise at improving blood glucose levels in individuals with type 2 diabetes: a randomised crossover trial	Diabetologia	https://doi.org/10.1007/s00125-018-4767-z
Schein et al., 2020	Acute inspiratory muscle exercise effect on glucose levels, glucose variability and autonomic control in patients with type 2 diabetes: A crossover randomized trial	Autonomic Neuroscience: Basic and Clinical	https://doi.org/10.1016/j.autneu.2020.102669
Schleh et al., 2020	Energy Deficit Required for Exercise-induced Improvements in Glycemia the Next Day	Medicine & Science in Sports & Exercise	https://doi.org/10.1249/MSS.0000000000002211
Scott et al., 2019a	Fasted High-Intensity Interval and Moderate-Intensity Exercise Do Not Lead to Detrimental 24-Hour Blood Glucose Profiles	The Journal of Clinical Endocrinology and Metabolism	https://doi.org/10.1210/jc.2018-01308
Scott et al., 2019b	High-Intensity Interval Training Improves Aerobic Capacity Without a Detrimental Decline in Blood Glucose in People With Type 1 Diabetes	The Journal of Clinical Endocrinology and Metabolism	https://doi.org/10.1210/jc.2018-01309
Sevilla-Lorente et al., 2025	Sexual dimorphism on the acute effect of exercise in the morning vs. evening: A randomized crossover study	Journal of Sport and Health Science	https://doi.org/10.1016/j.jshs.2024.101021
Shambrook et al., 2020	A comparison of acute glycaemic responses to accumulated or single bout walking exercise in apparently healthy, insufficiently active adults	Journal of Science and Medicine in Sport	https://doi.org/10.1016/j.jsams.2020.02.015
Solomon et al., 2020	Immediate post-breakfast physical activity improves interstitial postprandial glycemia: a comparison of different activity-meal timings	European Journal of Physiology	https://doi.org/10.1007/s00424-019-02300-4
Sparks et al., 2021	Alterations in Glycemic Variability, Vascular Health, and Oxidative Stress following a 12-Week Aerobic Exercise Intervention-A Pilot Study	International Journal of Exercise Science	https://doi.org/10.70252/CXSV8407

Tanaka et al., 2021	Effect of a single bout of morning or afternoon exercise on glucose fluctuation in young healthy men	Physiological Reports	https://doi.org/10.14814/phy2.14784
Tee et al., 2023	Combined effects of exercise and different levels of acute hypoxic severity: A randomized crossover study on glucose regulation in adults with overweight	Frontiers in Physiology	https://doi.org/10.3389/fphys.2023.1174926
Tee et al., 2024	Effects of exercise modality combined with moderate hypoxia on blood glucose regulation in adults with overweight	Frontiers in Physiology	https://doi.org/10.3389/fphys.2024.1396108
Toghi-Eshghi & Yardley, 2019	Morning (Fasting) vs Afternoon Resistance Exercise in Individuals With Type 1 Diabetes: A Randomized Crossover Study	The Journal of Clinical Endocrinology & Metabolism	https://doi.org/10.1210/jc.2018-02384
van Meijel et al., 2023	Effects of hypoxic exercise on 24-hour glucose profile and substrate metabolism in overweight and obese men with impaired glucose metabolism	American Journal of Physiology-Endocrinology and Metabolism	https://doi.org/10.1152/ajpendo.00230.2022
Wowdzia et al., 2022	Glycemic response to acute high-intensity interval versus moderate-intensity continuous exercise during pregnancy	Physiological Reports	https://doi.org/10.14814/phy2.15454
Yardley et al., 2019	Effects of Moderate Cycling Exercise on Blood Glucose Regulation Following Successful Clinical Islet Transplantation	The Journal of Clinical Endocrinology and Metabolism	https://doi.org/10.1210/jc.2018-01498
Yardley, 2020	Fasting May Alter Blood Glucose Responses to High-Intensity Interval Exercise in Adults With Type 1 Diabetes: A Randomized, Acute Crossover Study	Canadian Journal of Diabetes	https://doi.org/10.1016/j.jcjd.2020.09.007
Zaharieva et al., 2020	No Disadvantage to Insulin Pump Off vs Pump On During Intermittent High-Intensity Exercise in Adults With Type 1 Diabetes	Canadian Journal of Diabetes	https://doi.org/10.1016/j.jcjd.2019.05.015
Zhang et al., 2021	Walking Initiated 20 Minutes before the Time of Individual Postprandial Glucose Peak Reduces the Glucose Response in Young Men with Overweight or Obesity: A Randomized Crossover Study	The Journal of Nutrition	https://doi.org/10.1093/jn/nxaa420
Zhang et al., 2023	Effects of accumulated versus continuous individualized exercise on postprandial glycemia in young adults with obesity	European Journal of Sport Science	https://doi.org/10.1080/17461391.2023.2177199
Zheng et al., 2020	Effects of Exercise on Blood Glucose and Glycemic Variability in Type 2 Diabetic Patients with Dawn Phenomenon	BioMed Research International	https://doi.org/10.1155/2020/6408724
